# Pampean megamammals in Europe: the fossil collections from Santiago Roth

**DOI:** 10.1186/s13358-023-00283-5

**Published:** 2023-09-29

**Authors:** Damián Voglino, Jorge D. Carrillo-Briceño, Heinz Furrer, Ana Balcarcel, Gizeh Rangel-de Lazaro, Gabriel Aguirre Fernández, Analía M. Forasiepi

**Affiliations:** 1 Museo de Ciencias Naturales “A. Scasso” (Observatorio del Patrimonio Arqueológico Y Paleontológico OPAP, CRePAP, Dirección Provincial de Patrimonio Cultural), Calle Don Bosco 580, 2900 San Nicolás de los Arroyos, Buenos Aires Argentina; 2 Department of Paleontology, Universität Zürich, Karl-Schmid-Straße 4, 8006 Zurich, Switzerland; 3 Instituto Argentino de Nivología, Glaciología Y Ciencias Ambientales (IANIGLA), Universidad Nacional de Cuyo, Consejo Nacional de Investigaciones Científicas Y Técnicas (CONICET), CCT-Mendoza, Av. Ruiz Leal S/N° Parque Gral. San Martín, 5500 Mendoza, Argentina

**Keywords:** Quaternary, Fossils, Nineteenth century explorations, South America, Argentina, Europe, Cuaternario, Fósiles, Exploraciones del siglo XIX, América del Sur, Argentina, Europa

## Abstract

Santiago Roth was a Swiss fossil finder, naturalist, and paleontologist that emigrated to Argentina in 1866. His work largely influenced the discipline in the country at the end of the twentieth century, particularly the stratigraphy of the Pampean region. Some of his collections of Pampean fossils were sold to museums and private collectors in Europe and were accompanied by elaborated catalogues. Fossils in the Roth’s catalogues N° 2 and 3 are housed today in the Natural History Museum of Denmark, fossils from catalogues N° 4 to 6, were sold to Swiss museums, with Catalogue N° 5 currently housed at the Department of Paleontology, Universität Zürich. Here, we provide a general framework on the stratigraphy from the Roth’s Pampean fossil sites, summarize the history of the Pampean fossils in Europe originally collected by Roth, and provide historical and curatorial details of the Roth’s collection at the Department of Paleontology, Universität Zürich.

**Supplementary Information:**

The online version contains supplementary material available at 10.1186/s13358-023-00283-5.

## Resumen

Santiago Roth fue un buscador de fósiles, naturalista y paleontólogo suizo que emigró a la Argentina en 1866. Su obra influyó en gran medida en la disciplina del país a fines del siglo XX, con algunos aportes que son pilares, en particular aquellos para la comprensión de la estratigrafía de la Región Pampeana. Algunas de sus colecciones de fósiles pampeanos fueron vendidas a museos y coleccionistas privados en Europa y estaban acompañadas de catálogos elaborados. Los fósiles de los catálogos N° 2 y 3 de Roth se encuentran hoy en el Museo de Historia Natural de Dinamarca, los fósiles de los catálogos N° 4 a 6 fueron vendidos a museos suizos, mientras que el Catálogo N° 5 en particular está alojado actualmente en el Departamento de Paleontología, Universidad de Zurich. Aquí proporcionamos un marco general sobre la estratigrafía de los sitios de los fósiles pampeanos de Roth, resumimos la historia de los fósiles pampeanos en Europa colectados originalmente por Roth y brindamos detalles históricos y curatoriales de la colección de Roth del Departamento de Paleontología, Universidad de Zurich.

## Introduction

Santiago Roth was a Swiss naturalist and fossil finder who emigrated to Argentina in 1866 at the age of 16 (Bond, 1999; Fernández, 1925; Machon, 1925; Sánchez-Villagra et al., 2023; Weigelt, 1951). Soon after he and his family settled in the township of Baradero, north east of Buenos Aires Province, he began collecting fossils in the vicinities of his new home. The Pampean region of southern South America was first made famous in the academic world by the discovery of the giant ground sloth *Megatherium americanum,* that was sent to Spain and originally studied by Cuvier (1796). Later in the early nineteenth century, the voyages of discovery of the renowned naturalists Alcides D’Orbigny and Charles Darwin provided science with several new fascinating extinct creatures from this area, some of these influencing the formulation of the theory of evolution (Darwin, 1859; see Lister, 2018). Roth, in the late nineteenth–early twentieth centuries, continued this legacy and his work ultimately resulted in detailed geological descriptions and fossil collections that significantly contributed to the paleontology, geology, and biostratigraphy of the Pampean region. Since then, the area resulted in new discoveries and profound studies, including systematics, taphonomy, geochronology, magnetostratigraphy, biostratigraphy, isotope analyses, paleoproteomics, ancient DNA work, paleoecology, and paleoenvironmental reconstructions (e.g., Cione & Tonni, 1999, 2005; Delsuc et al., 2016, 2019; Domingo et al., 2020; Fariña et al., 2013; Metcalf et al., 2016; Pascual, 1966; Prevosti et al., 2021; Westbury et al., 2017). Fossils from the area are in Museo Argentino de Ciencias Naturales “Bernardino Rivadavia” and Museo de La Plata in the main cities of Buenos Aires and La Plata, respectively; in addition to the Museo Paleontológico “Fray Manuel de Torres” in San Pedro, Museo Municipal de Ciencias Naturales “Carlos Ameghino” in Mercedes, Museo Municipal “Casa de Ameghino” in Luján, Museo Municipal de Paleontología y Arqueología “José F. Bonaparte” in Salto, Museo de Ciencias Naturales “Lucas Kraglievich” in Marcos Paz, Museo de Ciencias Naturales “P. Antonio Scasso” in San Nicolás de los Arroyos (these latter in north east Pampean region, area that we focused in the text), Museo Municipal de Ciencias Naturales “Pachamama” in Santa Clara del Mar, Museo de Ciencias Naturales de Miramar “Punta Hermengo” in Miramar, Museo de Ciencias Naturales "Dr. José Squadrone" in Necochea, Museo Municipal de Ciencias Naturales “Lorenzo Scaglia” in Mar del Plata, among several others institutions which promote local research in natural sciences and store rich collections of Pampean fossils that are constantly unearthed at riverbanks, sea coast, or during artificial land removal by human activities.

During Roth’s time in Argentina, newly discovered extinct species, such as megamammals from the Pleistocene, as well as presumed associated human remains were precious scientific specimens worldwide. Some of the fossil material collected by Roth was eventually sold to private collectors and museums in Europe (Bond, 1999; Sánchez-Villagra et al., 2023; Torres, 1927). Among them, six collections have been identified, some containing detailed information in catalogues written by Roth. Of these, two are currently housed at the Zoologisk Museum, København in Denmark (e.g., Hansen, 2019, 2020), and one at the Paläontologisches Institut und Museum in Zurich in Switzerland. Other Pampean fossils collected by Roth are today at the Muséum d’histoire Naturelle de Genève and the Musée Cantonal de Géologie Lausanne, both in Switzerland; however, until now, reliable information about their catalogues is unknown.

The geological–paleontological work of Roth exceeds much beyond the Pampean region. He participated in several expeditions through Argentina, of which the Patagonian expeditions particularly enriched the collections of the newly founded Museo de La Plata. The MLP was inaugurated in 1888 with Francisco Pascasio Moreno as the first director. Moreno hired Roth as leader of the paleontological department in 1895 (Torres, 1927), the same year Roth started collecting fossils for the museum in Patagonia (Simpson, 1984). In 1895/1896, Moreno commissioned Roth as geologist and paleontologist in a trip to Patagonia (including the area of the Río Negro, Limay, and Collón Cura rivers, and Nahuel Huapi Lake, in Río Negro and Neuquén provinces) and this was the start of a series of missions in which Roth worked as member of the MLP. Results of that expeditions include the discovery of important localities and rich fossil associations, with exquisite specimens from the Paleogene and early Neogene. These include the Middle Miocene site at the Collón Cura River (Neuquén Province) and correlating levels at Río Negro and Chubut provinces, the Eocene fauna at Cerro del Humo (Roth’s “Cretáceo Superior Lago Musters”), the Early Oligocene fossils from Cañadón Blanco (Chubut Province), among several others (e.g., Roth, 1899, 1901, 1903; see also Reguero, 1998; Sánchez-Villagra et al., 2023; Simpson, 1936).

**Fig. 1:**
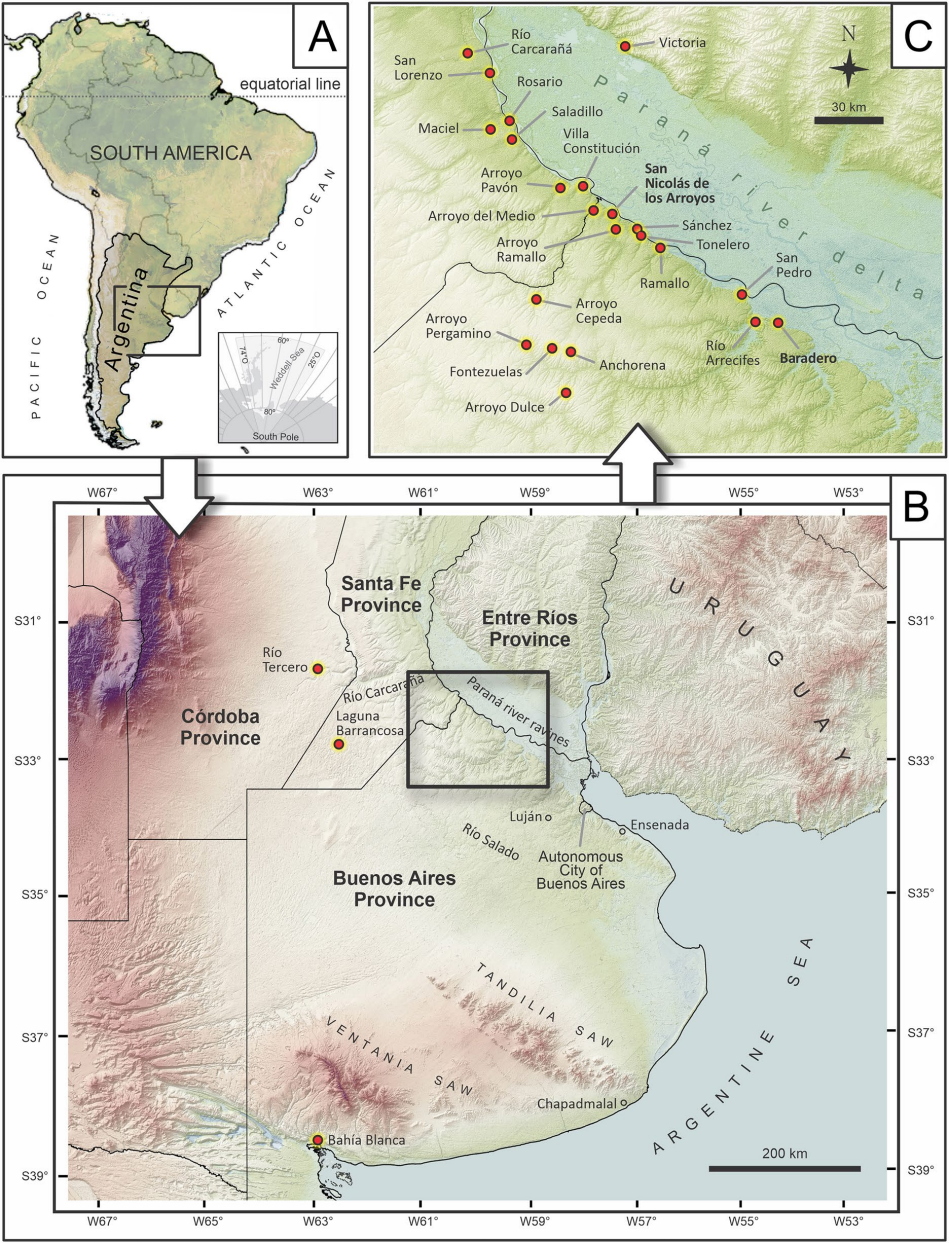
Geographical location of the fossiliferous sites mentioned by Roth in his catalogues. The sites are in Pampean region, Argentina (**A**), including Buenos Aires, Córdoba, Entre Ríos, and Santa Fe provinces (**B**). Localities are not exact. Detail of the sites at the riverbanks of the Paraná River are shown in (**C**). Map produced with QGIS V.3 3.22.0. The shape for the map was obtained from Instituto Geográfico Nacional, Argentina (http://www.ign.gob.ar)

In this contribution we focus on the collections that Roth retrieved from the Pampean region (Fig. 1), and that were acquired by institutions in Europe, and in particular the one housed in Zurich (PIMUZ). We provide a general framework of the stratigraphy at the Pampean sites, where fossils were unearthed (with the limitations and uncertainties due to more than a century passing since their original finding), summarize the history of the Pampean fossils in Europe that were originally collected by Santiago Roth, and provide historical and curatorial details of the Roth collection at PIMUZ.

### Institutional abbreviations

MACN, Museo Argentino de Ciencias Naturales “B. Rivadavia”, Ciudad Autónoma de Buenos Aires, Argentina (PV, vertebrate paleontology collection); MCGL, Musée Cantonal de Géologie Lausanne, Switzerland; MHNG, Muséum d’histoire Naturelle de Genève, Switzerland; MLP, Museo de la Plata, Buenos Aires, Argentina; PIMUZ, Palaeontological Institute and Museum of the University of Zurich, Switzerland; ZMK, Zoologisk Museum, København, Denmark.

### Other abbreviations

MBR, Matuyama–Brunhes geomagnetic reversal; MIS, Marine Isotope Stage; OIS, Oxygen Isotope Stages; OSL, Optically Stimulated Luminescence dating; US, sedimentary units (by its Spanish abbreviation “Unidades Sedimentarias” see Voglino & Pardiñas, 2005).

## Stratigraphy from Pampean sites and Roth’s legacy

The sedimentary deposits of the Pampean region are characterized by their marked lithological homogeneity, represented by reddish-brown sediments mainly composed of volcaniclastic sandy silts and silty sands, aeolian in origin (loess), with intercalation of paleosoils and calcrete (e.g., Fidalgo et al., 1975; Zárate & Blasi, 1991). D'Orbigny (1842) was the first to refer to them jointly as “terrains pampéenes” or “argiles pampéenes”, while later, Darwin (1845) used the name of the “pampean formation”. Since the end of the nineteenth century these names or similar alternatives have been used in the scientific literature, such as the “formación pampa”, “formación pampiano”, or “pampean sediments” (see summary in Prado et al., 2021; Tonni, 2011 and references therein). Roth, in his contributions, used the derivatives “formación pampeana” or “Pampasformation”. These early authors and other utilized the terms “formación” and “piso”, but their significance is different from the current understanding in lithostratigraphical and chronostratigraphical schemes (Tonni, 2011; in this later case stratigraphic units are in upper case).

**Fig. 2:**
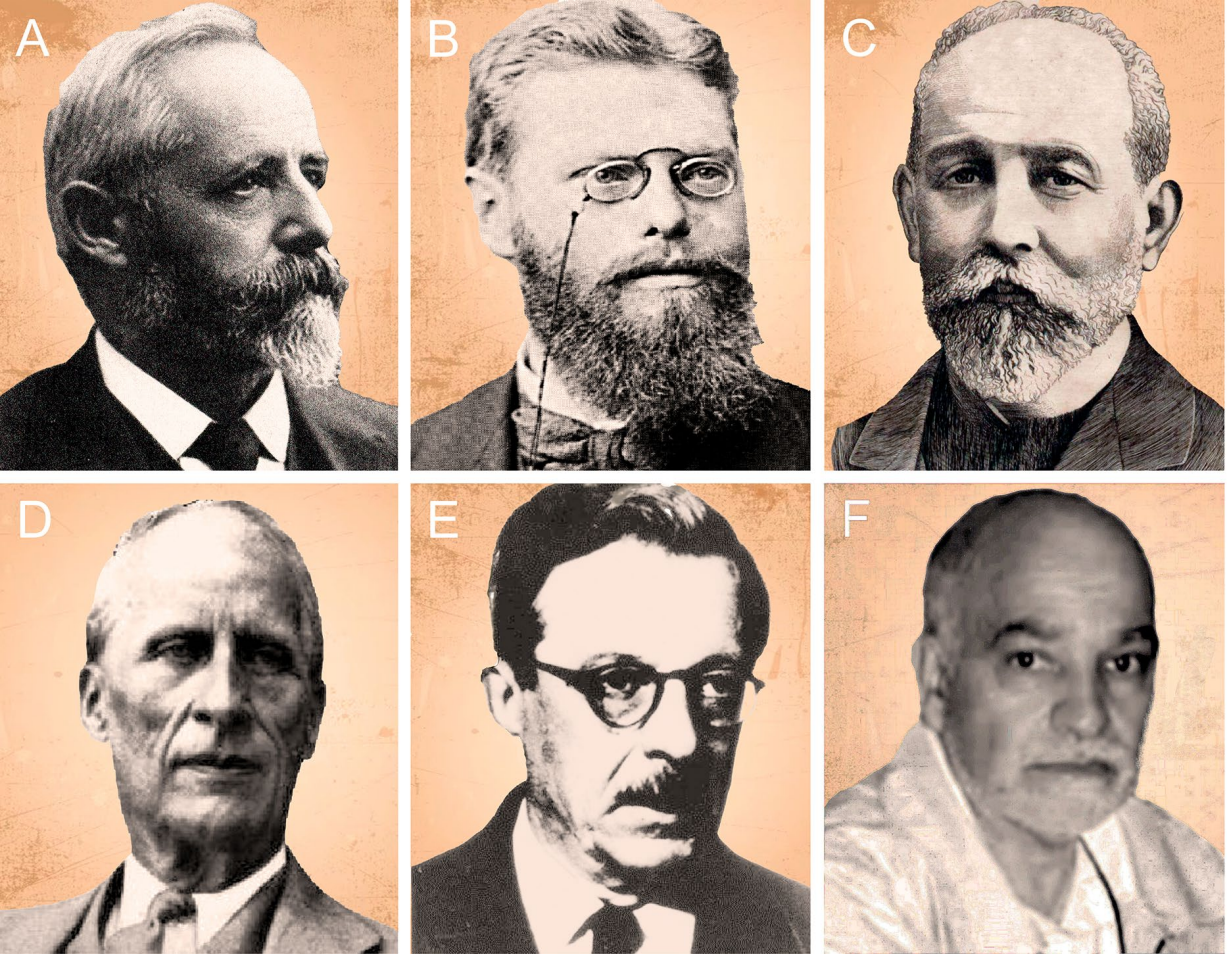
Some personalities that represents the pillars for their contribution to the studies on geology, stratigraphy, paleontology, and biochronology from the Pampean region. From left to right: Santiago Roth (**A**), Alfred Doering (**B**), Florentino Ameghino (**C**), Carl Burckhardt (**D**), Joaquín Frenguelli (**E**), and Eduardo Tonni (**F**). Images **A**, **D**, and **E** modified from Riccardi (2011); **B** from Tonni (2021); **C** drawing by R. Veroni, 1943 (archive from MACN-PV)

The extensive work of Roth in the Pampean region covered at least 25 localities in the Buenos Aires, Santa Fe, Córdoba, and Entre Ríos provinces (Voglino, 2020; Fig. 1; see also Carrillo & Püschel, 2023; Carrillo-Briceño et al., 2023; Le Verger, 2023; Ruiz-Ramoni et al., 2023). From these, the most profusely studied were sites in the neighborhood, where he lived (Baradero, Pergamino, and San Nicolás de los Arroyos—San Nicolás for brevity in the rest of the text) in the north east area of Buenos Aires Province (Torres, 1927). Roth performed several prospecting outings, made observations, and collected material at riverbanks of the Paraná River, including in San Lorenzo, Rosario, and Villa Constitución (Santa Fe Province), San Nicolás, Ramallo, San Pedro, and Baradero (Buenos Aires Province). He also extended this prospecting to tributaries (rivers and streams), such as the Río Carcarañá, Arroyo Pavón, Arroyo del Medio, Arroyo Ramallo, and Río Arrecifes. The Arroyo del Medio locality (Fig. 1) is between Buenos Aires and Santa Fe provinces, but the exact location on the side of the river, where Roth collected each fossil under this name is unknown. Few contemporaneous academics or those who followed (e.g., Florentino Ameghino, Carl E. Burckhardt, Joaquín Frenguelli, Alfredo Castellanos; Fig. 2) performed the systematic paleontological work focused on this area of the Pampean region as deeply as Roth. This situation changed in the late twentieth century with a steep increase in research covering different disciplines (see below).

**Fig. 3:**
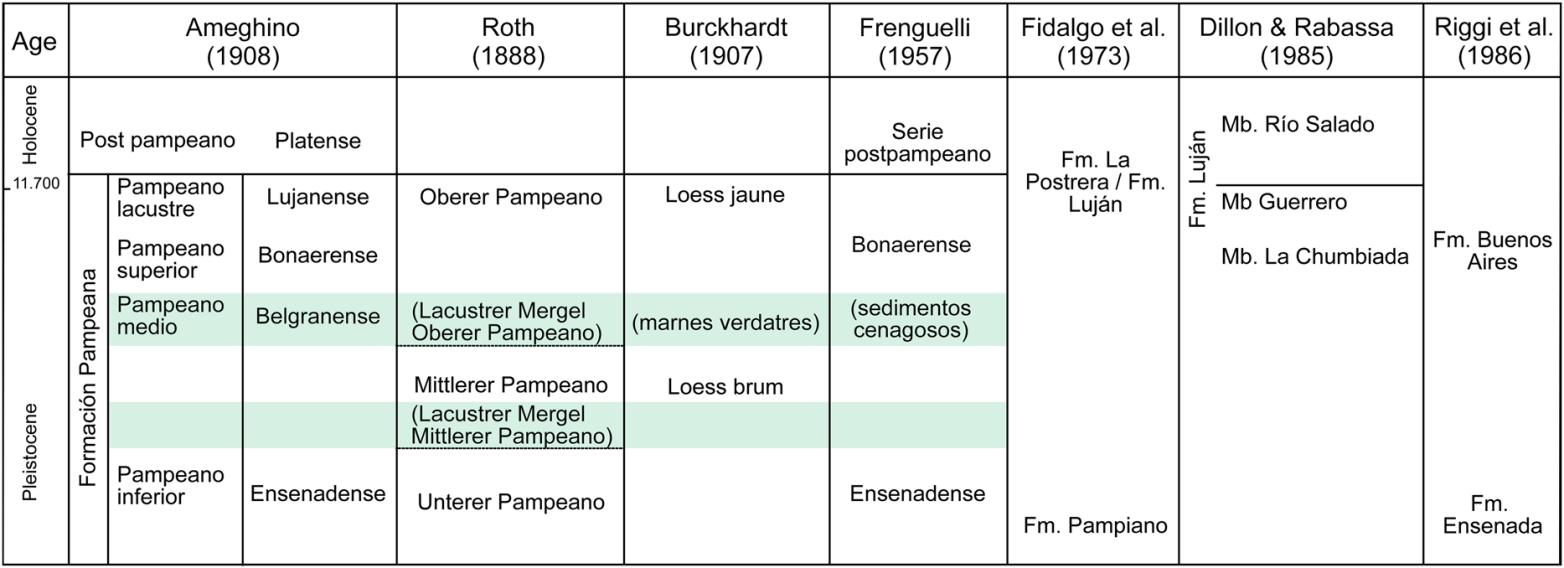
Principal stratigraphic schemes for the Pampean region

The stratigraphic studies by Roth in the Pampean region were primarily based on the scheme of Adolf Doering (1882) and Florentino Ameghino (1881, 1889, 1908) (Fig. 2B, C). According to Ameghino (1881), the “pampean formation” was divided into three units: “lower pampean”, “upper pampean”, and “lacustrine pampean”; these being overlayed by the “postpampean” (summary in Tonni, 2011). Later, and based on the geological studies of Doering (1882), Ameghino (1889) further divided the “pampean formation” in “pisos”: the “ensenadense”, characterized by the fauna recovered in the sediments from “La Ensenada” during the excavations for the construction of the La Plata harbor. This was followed by the “piso pampeano superior” or “bonaerense” and “piso pampeano lacustre” or “lujanense”, characterized by the fauna from the Luján River. He also documented a marine level between the “ensenadense” and “bonaerense” exposed along the coast of the Río de La Plata and Paraná River that received the name “piso pampeano medio” or “belgranense” (Ameghino, 1889; Fig. 3). Inland, this level was associated with a continental “belgranense” (see Tonni, 2011, and references therein).

**Fig. 4:**
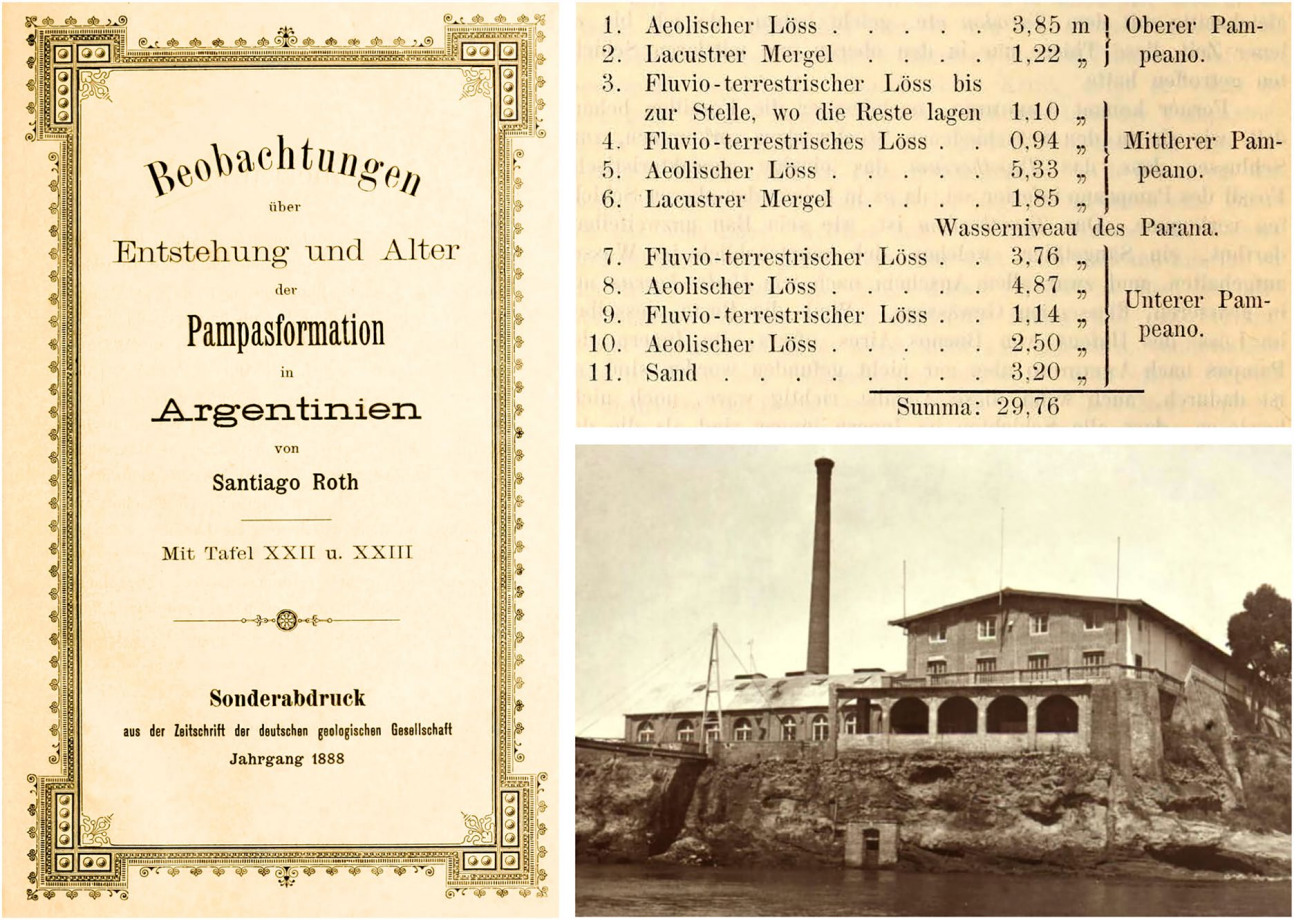
Cover of one of the most influential works by Roth (1888a) with his fundamentals on the stratigraphy of the Pampean region (left). This contribution contains the stratigraphic section from the Paraná riverbank (top right), today crucial information to interpret Roth´s ideas and the major divisions of the "pampean formation" (Roth, 1888a: 404). Photograph of “La Elisa”, the first slaughterhouse in South America, built in 1882 in the town of San Nicolás de los Arroyos (photograph from the archives of the Museo y Archivo Histórico “Gregorio Santiago Chervo”) (bottom right). The excavations for the construction of “La Elisa” benefited access to the stratigraphical sections of the riverbanks of the Paraná River

Based on observations at the riverbank of the Paraná River and tributaries, Roth (1888a) divided the “pampean formation” into four levels. From older to younger: (1) Untere Pampasformation (= pampeano inferior, in the Spanish literature, or lower pampean, in this contribution); (2) Mittlere Pampasformation (= pampeano intermediar or intermediate pampean); (3) Obere Pampasformation (= pampeano superior or upper pampean), and (4) Humusschicht (= terreno humus or humus layer) (Figs. 3, 4). Roth used this scheme in the catalogues that accompanied the fossils sold in Europe, as well as the collections housed in Argentina. However, this scheme was extensively criticized by Florentino Ameghino. To his view (Ameghino, 1908), Roth introduced several modifications (e.g., Roth, 1888a, 1908) to the stratigraphic scheme originally proposed by him that were consequences of extrapolations from original observations at the banks of the Paraná River to other more distant regions in the Chaco-Pampean region. A particular controversy was that Ameghino (1908) did not accept the inclusion of older strata, such as his own “piso hermosense” (“hermósico” from Ameghino, 1889) in the “lower Pampean” as well as other units previously considered “prepampeanas”, as Roth (1888a, 1908) did. “*No me es posible continuar con el examen del terreno y la exposición de mis observaciones, sin aclarar ante todo lo que se refiere á la nomenclatura, de la cual han hecho un verdadero galimatías. En esos trabajos se habla del pampeano inferior de Ameghino y del pampeano inferior de Roth; de las capas de Monte Hermoso según el sistema de Ameghino y del pampeano inferior é intermedio según el sistema de Roth; de correlaciones entre los horizontes establecidos por uno de esos autores con nombres definidos, con los establecidos por el otro con los mismos nombres; se refiere el hermosense al pampeano inferior con el cual no tiene absolutamente nada que ver, *etc*., *etc*. Una confusión espantosa en la cual no tengo ni culpa ni parte*” (Ameghino, 1908: 359). The spirit of this discussion also reflected previous conflicts between both scientists (e.g., Roth, 1894), stressing the confrontation with the Museo de La Plata from which Florentino Ameghino had resigned in 1887 (Simpson, 1984). Nevertheless, they agreed on both models of the “lower Pampean” corresponding to the lower portion of the “pampean formation” above the “puelchense” (= Puelches Formation) due to the shared presence of a fossil mammal of stratigraphic significance: the mid-sized and extinct native ungulate, *Mesotherium cristatum.* By Ameghino’s time, this creature was known as “*Typotherium*”, based on the name given by Bravard (1857; *Typotherium medium* and *Typotherium minutum,* both *nomina nuda*; Mones, 1986); however, the species was first described and later formally nominated as *Mesotherium cristatum* by Serrés (1867; see also Tonni, 2011; Fernandéz-Monescillo et al., 2023). *Mesotherium cristatum* is a characteristic extinct mammal from the Lower and Middle Pleistocene of the Pampean region (Cione &Tonni, 2005).

As mentioned above, the riverbanks of the Paraná River were the stratigraphic key reference for Roth and the basis for explaining his interpretations of the divisions of the "pampean formation" (Roth, 1888a). He took advantage of excavations for the construction of the slaughterhouse “La Elisa” in 1882 to describe the stratigraphic sections exposed at the riverbanks in San Nicolás (Roth, 1888a; Fig. 4). His detailed descriptions associated with his stratigraphic scheme make this section essential to understanding the provenance of many of the specimens included in the collection catalogues. In recent works (Voglino, 2020), this stratigraphic section has made it possible to update stratigraphic correlations in the Pampean region. Roth included a greenish silty-clay, lacustrine level (= Lacustrer Mergel) in the “upper Pampean” and likely used it, along with associated paleosoils, as marker beds to locally benchmark the origin of the fossils he collected. However, he also emphasized that these deposits were intermittent and cannot be used to delimit formal units (i.e., “formations”; Roth, 1888a: 399). He challenged the ideas of Ameghino emphasizing that lacustrine deposits per se lacked stratigraphic significance, since in the area of the Luján River, these sediments are in the “upper Pampean”, in the locality of San Lorenzo they appear in the “lower Pampean”, while in San Nicolás they are frequently in the “intermediate Pampean”. Later authors (e.g., Castellanos, 1938; De Carles, 1912; Frenguelli, 1946) also followed Roth’s lacustrine level (Fig. 3).

Later, and based on the color of sediments, Carl Burckhardt (1907) (Fig. 2D) suggested a different scheme, dividing the “pampean formation” into the “loess brun” and “loess jaune” (Fig. 3). In addition, Burckhardt emphasized that greenish levels (“marnes verdatres”) intercalate between his divisions of the “pampean formation” and recognized an erosive surface at the top of the “loess brun” that could be used as a guide horizon between both of his units …“*division qui a été proposée pour la première fois par M. Roth, l'explorateur bien mérité de la formation pampéenne*” Burckhardt, 1907: 151).

Joaquín Frenguelli (1925, 1946) (Fig. 2E) based on his studies at the Paraná River in Rosario and utilized the terms “piso bonaerense” for the loess at the top of the riverbank and “piso ensenadense” for the basal and middle exposed silts, with the intercalation of greenish sediments—his “sedimentos cenagosos” (Fig. 3). The “belgranense” from previous authors was included in his “ensenadense”. In Frenguelli’s concept, Roth’s “lower” and “intermediate Pampean” corresponded to his “ensenadense”, while the “sedimentos cenagosos” (= Lacustrer Mergel from Roth = marnes verdatres from Burckhardt) represented the boundary between the “ensenadense” and the “bonaerense”. A similar scheme was followed by Castellanos (1938) who used greenish marls, his “margas verdosas”, to separate the units (see Voglino, 2020 for clarifications).

Since the mid-twentieth century deposits from the Pampean region received vast attention. Disputing proposals attribute the late Neogene Pampean sediments to a single stratigraphic unit or subdividing them into different schemes. One of the most frequent stratigraphic schemes suggests division of the sedimentary deposits into the Ensenada, Buenos Aires, Luján (including the La Chumbiada, Guerrero, and Río Salado members), and La Postrera formations (e.g., Dillon & Rabasa, 1985; Fidalgo et al., 1973, 1975; Riggi et al., 1986; Tonni et al., 1999; Zárate & Blasi, 1991) (Fig. 3). Several of these studies based their analyses on other localities from the Pampean region, mainly focusing on the Atlantic coast. In contrast, the area originally investigated by Roth in the central east area of Argentina was neglected by the academic community, despite its renown and profound impact on local paleontology and stratigraphy. It was only during the last decade of the twentieth century that Roth’s area at the riverbanks of the Paraná River and tributaries was focused on again in subsequent studies, integrating the stratigraphy from north Buenos Aires and Santa Fe (e.g., Fucks & Deschamps, 2008; Iriondo & Kröhling, 1995, 2009; Irrazabal & Rey, 2015; Kröhling, 1996, 1999a, 1999b; Nabel et al., 1993, 1999; Parent & Vega, 2005; Parent et al., 2003; Tófalo et al., 2008; Toledo, 2009, 2011; Voglino, 2008, 2020; Voglino y Pardiñas, 2005).

Today, the outcrops in Roth’s collecting area are referred to as the “pampean formation” or informally as “sedimentos pampeanos” and constitute part of the Ensenada Formation and the Buenos Aires Formation (Fig. 3). These units are representative of the Pleistocene and were used by many authors, but it was Riggi et al. (1986) who described and formally defined them (Tonni, 2011; Tonni et al., 1999).

### Chronostratigraphic/geochronological scale for the Pampean region

**Fig. 5:**
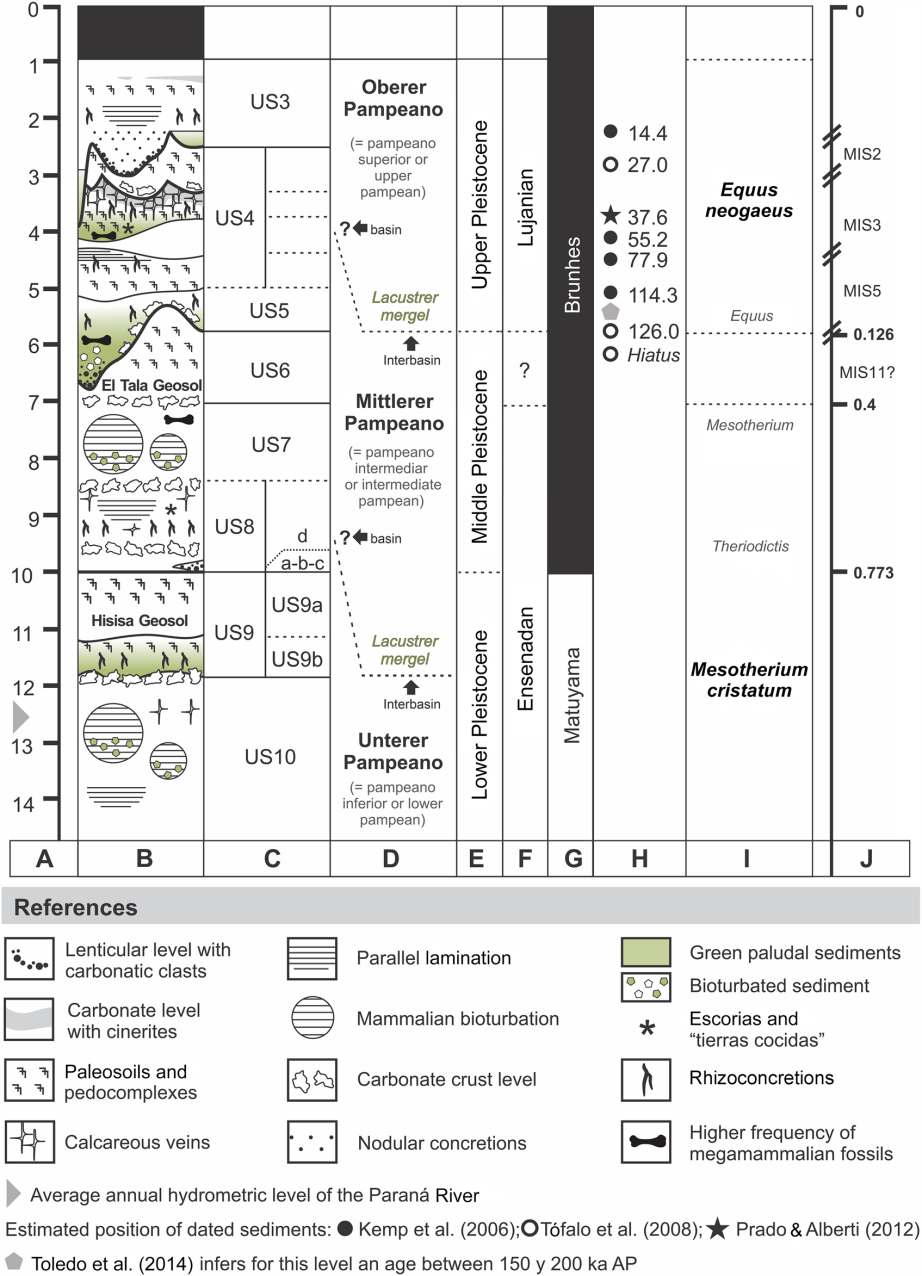
Comprehensive stratigraphic section from the Paraná River (north of Buenos Aires and south of Santa Fe provinces), geochronological, chronostratigraphic, and biostratigraphic references based on previous studies in the locality of Ramallo (Buenos Aires Province; Voglino & Pardiñas, 2005). This section was used to interpret the stratigraphic scheme proposed by Roth (Voglino, 2020). References: **A** Depth meters of the stratigraphic section. **B** Squematic stratigraphic section from the Paraná riverbanks; **C** Sedimentary Units (US, by its abbreviation in Spanish) based on Voglino and Pardiñas (2005); **D** Roth’s stratigraphic scheme for the “pampean formation”; **E** Epochs; **F** South American Stages/Ages; **G** Magnetic polarity; **H** TL and OSL dating (black circle: Kemp et al., 2006; white circle: Tófalo et al., 2006; star: Prado & Alberdi, 2012); **I**, Biozones (Cione et al., 2015); **J**, Marine Isotopic Stages

The extensive work of Eduardo P. Tonni (Fig. 2F) and colleagues (e.g., Cione & Tonni, 1995, 1999, 2001, 2005; Tonni et al., 1992, 1999, and subsequent contributions; Cione et al., 2007, 2015, and others) focused on integrating the broad stratigraphic, radiometric, and paleomagnetic information with the paleontological record. They proposed a chronostratigraphic/geochronological scale for the Pleistocene–Holocene of the Pampean region adjusting the concepts of the Ensenadan, Bonarian, Lujanian, and Platan to Stages/Ages, based on biozones (Fig. 5). The comprehensive scheme is as follows (temporal limits or taxa characterizing the biozones is currently under debate; e.g., Fernández-Monescillo et al., 2023; Toledo et al., 2014): the *Mesotherium cristatum* Biozone characterizes the Ensenadan Age/Stage (Lower to Middle Pleistocene; 1.78–0.4 Ma). This biozone correlates with the Ensenada Formation in the Pampean region (e.g., Cione et al., 2015). The *Megatherium americanum* Biozone is the base for the Bonaerian Stages/Ages (Middle Pleistocene; 400–126 ka). Its lower limit correlates with the base of the Buenos Aires Formation (e.g., Cione et al., 2015). This biozone began in the interglacial period corresponding to MIS11, about 0.4 Ma (Cione & Tonni, 2001; Cione et al., 2015; Prado et al., 2021; Verzi et al., 2004). The *Equus neogaeus* Biozone is the biostratigraphic base for the Lujanian Stages/Ages (Upper Pleistocene; 126–7 ka). This unit includes the interglacial period MIS5e (ca.125 ka) or MIS3 (ca. 57 ka), the Last Glacial Maximum (26–20 ka) and the Younger Dryas (12.900–11.700), as well as the first record of humans in the Pampean region (e.g., Cione et al., 2015; Prado et al., 2021). The *Lagostomus maximus* Biozone is the base for the Platan (Holocene; 7 ka–1492 AD). Its base correlates with the Río Salado Member of the Luján Formation and also includes the La Postrera Formation. The end of this biozone is marked by the fauna introduced by Europeans (Cione et al., 2015).

## The legacy of Roth revisited

Recent fieldwork in the Pampean region by Voglino and Pardiñas (2005) and Voglino (2020) allowed interpretations of the original descriptions provided by Roth and correlations with current stratigraphic schemes. These contributions provided a comprehensive section (Fig. 5), representative for the whole region at the riverbanks along the Paraná River (Fig. 1), which allowed correlations with neighborhood areas, considering the abrupt local changes and facies.

At Ramallo, Voglino and Pardiñas (2005, see also Ruiz-Ramoni et al., 2023; Voglino, 2020) described a stratigraphic section of ca. 15 m dominated by sandstones at the base and siltstones at the top. There are frequent laminar eye-lens, calcareous concretions, paleosoils, paleoburrows, and fossil vertebrate remains. This section was divided into ten sedimentary units (US) labeled 1 to 10 from the top to the base (Fig. 5).

The US10 is exposed at the base of the riverbank of the Paraná River and consists in massive silt deposits with a level of large calcareous crusts and nodules at the top. During the ordinary and extraordinary floods of the river, this unit is submerged under the water.

The US9 is frequently seen at the base of the exposed riverbanks of the Paraná River and major tributaries. This unit contains a paleosoil level, broadly extended over the region and was correlated with the Hisisa Geosol (Nabel et al., 1990, 1993, 1999, 2000, originally described for the Ensenada Formation, in the area of San Pedro and Baradero). The Matuyama–Brunhes geomagnetic reversal event (MBR) dated 0.773 Ma has been originally recognized in the localities of San Pedro y Baradero above the Hisisa Geosol (see Nabel, 1993; Nabel et al., 1990), representing a relevant magnetostratigraphic marker for this area of sedimentological homogeneity (see also Tonni et al., 1999). Stratigraphic correlations are the basis for interpretation of the section exposed in Ramallo (Fig. 5; see also Voglino & Pardiñas, 2005). The base of the US9 unit (the US9b level) is characterized by greenish clayey silts (paludal sediments). Because of the notoriety of these deposits in the field and in Roth’s description of the Paraná riverbank stratigraphic section (Roth, 1888a: 404), we interpret that this level was likely used by him to divide the locally the “inferior Pampean” from the “intermediate Pampean”.

The US8 and US7 are represented by massive silts with occasional trough cross-bedding stratification. Fossil mammals of biostratigraphic significance, characteristics of the Ensenadan Stage/Age, such as *Mesotherium cristatum* and *Theriodictis platensis* (e.g., Prevosti & Palmqvist, 2001; Ruiz-Ramoni et al., 2023), were collected from this unit. Paleoburrows may sometimes contain fossils from overlying units and produce taphonomic modifications, including alterations in the stratigraphic sequence and mixture of fossils. Roth and Ameghino discussed the stratigraphic provenance of *Mesotherium cristatum,* the first supporting the view that this taxon was present not only in the “lower” but also in the “intermediate Pampean”. Our field observations support Roth’s interpretation, since some of new findings have been collected from US7. The following passage also reflect the debate between both paleontologists and long controversies: “*Antes afirmaba Ameghino, que sólo se encontraba el Typotherium en el pampeano inferior. Por eso tuve con él una explicación, y le dije que estaba equivocado, si creía que el Typotherium es el fósil característico del pampeano inferior, pues que yo había hallado ya restos de él en el pampeano intermediario. Al mismo tiempo, le mostré los restos del Typotherium Lausenii. Los reconoció en el acto como pertenecientes á una especie nueva que pudiera encontrarse en capas más nuevas, y no se quiso dar por convencido de que también se encuentra el Typotherium cristatum en capas más modernas. Más tarde, cuando encontré otro cráneo en las cercanías de San Nicolás, en la formación pampeana intermediaria, traté de desengañarlo; parece que reconoció su antiguo error; por lo menos designa como localidad del Typ. crist. San Nicolás y como capa, el piso belgranense, el cual á su modo de ver, es más reciente que las toscas del fondo del Río de la Plata*. (Roth, 1894: 20). In fact in his work of 1889, Ameghino included this taxon in his “pampeano inferior” and also in his “pampeano medio” or “belgranense” (Fig. 3); however, later Ameghino (1908: 361) omitted reference to Roth’s observations, dismissing the contradiction: “*El Typotherium cristatum que paleontológicamente caracteriza el «pampeano inferior» de Buenos Aires en el sentido de Ameghino, se encuentra en las mismas capas hasta San Nicolás, en donde es característico del «pampeano inferior» en el sentido de Roth*.”

The US6 includes a second paleosoil level of regional continuity, likely correlated with the El Tala Geosol studied in San Pedro and Baradero (Nabel et. al., 1993; Tonni et al, 1999). Following Tófalo et al. (2008), the US6 is similar to the pedogenetic event included within their unit A, that can be correlated with MIS9 or, more possibly MIS11 (Fig. 5). The already mentioned presence of *Mesotherium* at the top of US7 and the unconformity that separates this level from the overlaying US6 and US5, suggest that sediments potentially assignable to the Buenos Aires Stage/Age are poorly represented or absent in the study area. This interpretation agrees with the ideas of Tófalo et al. (2008) who recognized at the base of the riverbanks of the Paraná River in Zárate a discontinuity surface and a prolonged hiatus between their units A and B. An alternative was proposed by Toledo (2009), who interpreted this hiatus as neotectonic activity occurred ca. 500,000 years ago. The length of this process is unknown, but it is supposed to be prolonged, possibly between 700,000 and 130,000 years, during which the sedimentary record associated with MIS8 to MIS14 could have been eroded (Toledo, 2009). The distinct erosional unconformities between US7 and US6, as well as between US6 and US5 represent long temporal gaps; in addition, paleoburrows, paleochannels, and transported material are frequent in these units and indicate that stratigraphic interpretations have to be accompanied by detailed taphonomic studies and associated with biochronologically informative fossils.

The US5 is characterized by greenish clayey silts (paludal sediments) that overlays the US6 by an erosive unconformity. The US5 extends laterally interrupted over more than 200 km between Rosario (Santa Fé) and Campana (Buenos Aires). In Baradero, Kemp et al. (2006) studied paludal and eolian sediments altered by pedogenetic processes, that are probably correlated with our US5. The deposits located towards the base of the sequence provided an OSL age of 114.30 ± 7.20 ka (Kemp et al., 2006) and were referred to the last interglacial, equivalent to the MIS5 (> ca. 80,000 years). As in the case of the US9b, due to its remarkable visibility, uniformity, and lateral continuity, we interpret that this level could have been used by Roth (1888a) as landmark between his “intermediate Pampean” and “upper Pampean”.

The US4 consists of a paleosoil sequence overlayed by a level of paludal sediments intercalated by eolian deposits. In the study area, the US4 is exposed in interfluvial areas and drainage basins close to the mouths of large streams. We interpret that US4 shares similarities with the Carcarañá Formation (Kröhling, 1999b), with alluvial and marshy facies cropping out in the drainage basin (Iriondo & Kröhling, 2007). The US4 can be also partially correlated with the already mentioned paludal and eolian sediments studied by Kemp et al. (2006) in Baradero, which were altered by pedogenetic processes formed ca. 80 ka and 25 ka ago. OSL datings at the locality of San Pedro from levels correlatable with the middle part of US4 produced ages of 30.94 ± 2.5 ka and 36.30 ± 2.4 ka (Toledo, 2009); and 41.554 ± 3.756 ka and 37.626 ± 4.198 ka (Prado & Alberdi, 2012). However, more recently, Toledo et al. (2014) considered the latter “anomalous”, in turn suggesting older ages, ranging between 150 and 200 ka that were obtained from an isolated tooth of *Toxodon* sp. and associated microsparite grains. The notable discrepancies between the values obtained by different authors from the likely same level stress the need of deeper studies. The stratigraphy in the area is complex and characterized by abrupt lateral variation, including important hiatuses, paleorelief defined by undulations, erosive unconformities, bioturbation, and facies changes (Fig. 5). Similarly, fossils used for dating should be analyzed under rigorous taphonomic control. In the US4 bioturbation (e.g., crotovines) or paleochannels can alter the original position of the fossil material in the sequence and fossils may not be contemporaneous with the sediments that contain them.

Similarly, it is possible to correlate US4 with the succession of welded paleosoils present in the unit D and C of Tófalo et al. (2008) recognized in Zárate, which was formed towards the end of the last interglacial interval or the interstadial MIS 3 (Tófalo et al, 2008).

Additional stratigraphic markers in the area are friable sedimentary deposits, with high proportion of carbonates and cinerites, filling cracks. These levels lay over the paludal sediments and associated paleosoils of the US4, are few centimeters thick, and have little areal development (although present in several localities).

The US3 corresponds to the “loess jaune” from Burckhardt (1907) or the “loess” from Frenguelli (1925). It overlays the US4 by an erosive unconformity already mentioned by both authors, and is usually underlaying the present soil. This unit is characterized by friable silts with small subspherical concretions of calcium carbonate at the mid part of the unit. According to our interpretations, the US3 correlates with the upper levels of the Tezanos Pinto Formation (the “facies primaria” from Iriondo, 1980), attributed to aeolian accumulation under arid to semi-arid climatic conditions. Its age has been referred to the Late Pleistocene (MIS2). In the valleys, fluvial and alluvial facies were recognized with high concentration of fossils (Ferrero et al., 2019).

The complex of paleosoils and paludal sediments, intercalated with irregular or poorly defined limits that characterize US5 and US4, and also the basal levels of US3 (including its fluvial and alluvial facies), can likely be associated with the “belgranense” that several contemporary authors to Roth observed in the middle sectors of the riverbanks of the Paraná River (Fig. 3).

### Closing remarks

As indicated above, the levels of greenish clayey silts exposed at the riverbanks of the Paraná River, corresponding to our US9b and US5–US4 (and eventually at the base of US3) are outstanding features clearly visible in the stratigraphic sections and broadly distributed over a distance of more than 200 km. They constitute useful markers to interpret the stratigraphic origin of the fossil collections.

A sedimentary sequence similar to that exposed at the Paraná riverbanks is also observed in the mouths of tributary streams. However, when greenish clayey silt levels are exposed inland, these may correspond with other sequences characterized by a similar lithology, texture, and structure, but associated with a different stratigraphic scheme representative of valleys. In fact, some data provided by Roth contain misinterpretations. In his catalogues, for example, he assigned ages older than it should to taxa coming from streams and tributaries of the Paraná River. Toledo (2009) developed a detail analysis of the important historical consequences resulting from these confusions. As an example, a sedimentary level known as the “oyster bank”, associated with a marine transgression (Middle Holocene; Platan Stages/Ages), was discovered by Roth in San Pedro (Fig. 1). Initially, Roth attributed a pre-Quaternary age to these deposits, while Ameghino assigned to his “belgranense” (Fig. 3). Roth discovered human material in the vicinity of the “oyster bank” in the basin of the Río Arrecifes (Arroyo El Tala), Baradero (the “Baradero Man”; see Menéndez et al., 2023) while interpreting this finding as the likely oldest human skeleton from South America (Lehmann-Nitsche, 1907). This resulted in other contemporary researchers worldwide became interested in these deposits and fossils. However, more recent studies have demonstrated a much recent age for the archaeological material (< 5 ka years AP; Toledo, 2017; Toledo et al., 2010), based on Uth/ESR radiometric dating.

The stratigraphic correlation and the interpretation of provenance of the fossils in the study area is difficult not only because of the repetition of sedimentary units with similar lithology and structure, but also by the presence of marked erosion surfaces and temporal hiatus. In addition, some of the units (frequently US7, US8, and US9) have bioturbations caused by medium and large mammals (paleoburrows) (Fig. 5). The paleoburrows and crotovines from the Paraná riverbanks and tributaries were scarcely documented in previous references, despite the fact that in some areas they are very abundant. Similar structures were described by Imbellone and Teruggi (1988) and Imbellone et al. (1990) in other areas of the Pampean region. The paleoburrows and crotovines in the study area have cross section diameters ranging between 0.5 to more than 2 m. Like those described for the Atlantic coast (e.g., Cenizo et al., 2016; Zárate et al., 1998 and references therein), the structures are transgressive, crossing the stratigraphy discordantly (Vizcaíno et al., 2001), and filled by sediments of different ages despite the fact of having similar color and texture, thus being indistinguishable from the sediment around. In addition, the sediment extracted by the fossorial activity could form the frequent accumulations referred locally as diamicton, often with vertebrate remains. In short, these biological activities disturbed the original stratigraphy and mixed the sediments of different ages and their fossils. In our interpretation, considering the stratigraphic section presented by Roth (1888a) at San Nicolás and recent studies in the same area and vicinities of the Paraná River (Voglino, 2020), Roth’s units 1 and 2 (5.07 m, the Oberer Pampeano) correspond to the US3 to US5 from Voglino and Pardiñas (2005; Buenos Aires Formation; Lujanian Stage/Ages). Roth’s units 3 to 6 (9.22 m, the Mittlerer Pampeano) correspond to US6 to US9 from Voglino and Pardiñas (2005; Buenos Aires and Ensenada formations; Lujanian, likely Bonarian, and Ensenadan Stages/Ages), while Roth’s unit 7 (3.76 m, the Unterer Pampeano) correlates with US10 from Voglino and Pardiñas (2005; Ensenada Formation; Ensenadan Stages/Age). Our interpretation of the correlation of the stratigraphic levels as described by Roth (1888a) and the current biostratigraphic scheme for the Pampean region is indicated in Fig. 5.

## Roth collections at Europe

**Fig. 6:**
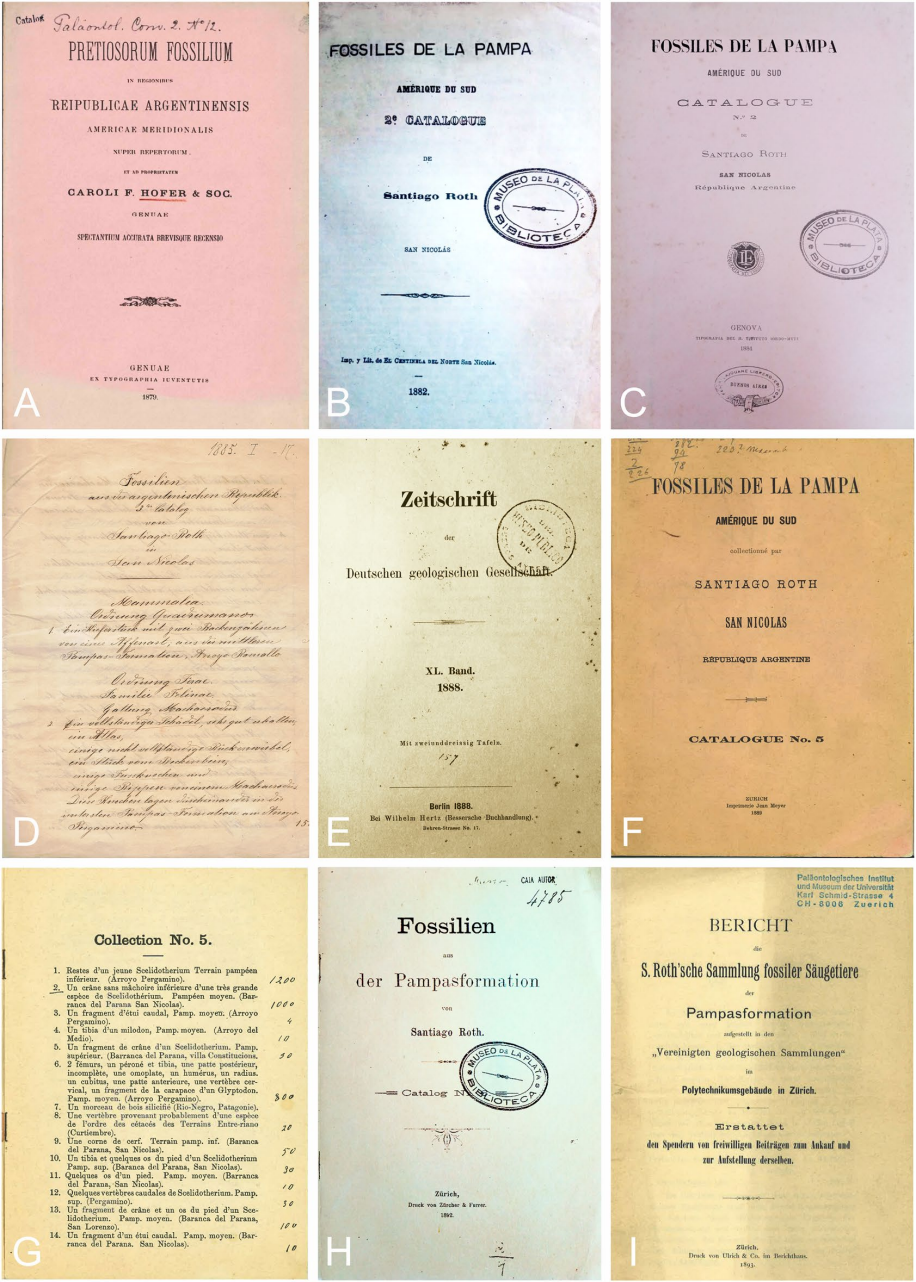
First pages of the catalogues from the Roth´s collections of fossil vertebrates from the Pampean Region. Catalogue Nº1 published in Genua in 1879 (**A**). Catalogue Nº2 published in San Nicolás in 1882 (**B**) and in Genua in 1884 (**C**). Catalogue Nº3, handwritten in San Nicolás in 1885 (**D**). Cover of the journal Zeitschrift der Deutschen Geologischen Gesellschaft with Catalogue Nº5 published in Berlin in 1888 (**E**). Catalogue Nº5 published in Zurich in 1889 (**F**) and first page of the catalogue, the numbers at the right in manuscript are the prices in Francs for each specimen (**G**). Catalogue Nº6 published in Zurich in 1892 (**H**). Public acknowledgment to the local Swiss community, several organizations and societies, and government for contribution to purchase Roth Catalogue N°5 (Heim & Lang, 1893) (**I**)

At the end of the nineteenth century, fossils from the Pampean region were very valuable objects for public institutions and private collectors. Santiago Roth recovered from the Pampean region hundreds of specimens most of them fossil megamammals, although some reptile and fish remains and archaeological material are also present in the collections. At least six chronologically numbered and partly printed catalogues were produced by Roth (Fernández, 1925; Machon, 1925), some dedicated to the fossil collections prepared for sale (Hansen, 2019). Catalogues include the following list (see also Fernández, 1925; Machon, 1925; Fig. 6):

*Catalogue N° 1*.

- Hofer, C.F. 1879. Pretiosorum Fossilium in regionibus Reipublicae Argentinensis. Americae Meridionalis. Nuper repertorum et ad proprietatem. Genua: Carolus F. Hofer & Soc. Pp. 8

*Catalogue N° 2*.

- Roth, S. 1882. Fossiles de la Pampa. Amérique du Sud. 2° Catalogue. San Nicolás. Buenos Aires: Imp. Y Lit. de El Centinela del Norte de San Nicolás. Pp. 12.
- Roth, S. 1884. Fossiles de la Pampa. Amérique du Sud. Catalogue N° 2. San Nicolás. République Argentine. Genova: Tipografía del R. Istituto Sordo-Mutti. Pp. 28.

*Catalogue N° 3*.

- Roth, S. 1885. Fossilien aus der argentinischen Republik. Catalog N° 3. San Nicolas (handwritten list).

*Catalogue N° 4*.

- Not accessible.

*Catalogue N° 5*.

- Roth, S. 1888. Zeitschrift der Deutschen Geologischen Gesellschaft. XL Band. Berlin: Wilhelm Hertz Bessersche Buchhandlung. Pp. 20.
- Roth, S. 1889. Fossiles de la Pampa. Amérique du Sud. Catalogue N° 5. Zurich : Jean Meyer.

Pp. 16.

*Catalogue N° 6*.

- Roth, S. 1892. Fossilien aus der Pampasformation. Catalog N° 6. Zurich: Zürcher & Furrer. Pp. 14.

Selling collections for a price was not an easy task for traders of fossils and other objects. Sellers often had to split up collections and sell parts separately. Written communications offering these collections went to all large museums in Europe, from Rome to Stockholm (Hansen, 2019; Weigelt, 1951). Some of the first collections for sale were presented to eventual buyers as handwritten lists (e.g., Roth, 1885: Catalogue N° 3; Fig. 6); however, others were printed in high-quality booklets, some with exquisite drawings (e.g., Roth, 1884: Catalogue N° 2), while other cases included individual specimen prices and a total sum for the whole collection (Hansen, 2019).

Among the paleontological collections that Roth sold in Europe, we highlight those acquired by Dr. Valdemar Lausen, which include all the specimens referred to in Roth Catalogues N° 2 and N° 3. These collections are currently housed at the Zoologisk Museum, København—ZMK (Hansen, 2019, 2020). Roth collection Catalogue N° 5 has a similar history. The government of Switzerland, the canton of Zurich, and private donators acquired the entire collection and is now housed at the Palaeontological Institute and Museum of the University of Zurich—PIMUZ (see below).

Dr. Valdemar Lausen (1834–1889) was a Danish medical doctor and philanthropist with great interest in paleontology (Hansen, 2020). He lived in Buenos Aires, where he bought fossil material from local fossil dealers. Lausen eventually donated his entire collection to the Zoological Museum Copenhagen (today ZMK), where the specimens are currently housed (Hansen, 2020).

In 1877 or 1878, Lausen purchased a first fossil collection from Roth (Hansen, 2019). According to Hansen (2020) the information supplied by Roth about this sale was scarce. Most specimens were labelled “Plata-landene” (in Danish), which roughly translates to “areas of land in the vicinity of the La Plata River” (Hansen, 2019, 2020).

In 1883 (Weigelt, 1951) or 1884, Lausen bought another collection that corresponds to the whole lot in Roth’s Catalogue N° 2 (Hansen, 2019). Catalogue N° 2 had two editions according to our search; one printed in San Nicolás (Roth, 1882) and the other in Genova (Roth, 1884). The first included prices, while the latter included exquisite drawings of the fossil skulls for sale. Catalogue N° 2 is organized taxonomically. The collection gathered 12 taxa that represent 101 catalogued specimens and included isolated remains, partial skeletons and skulls, and almost complete skeletons of mammals (including sabertooth felids, sloths, glyptodonts, notoungulates, horses). It also includes archaeological material such as an instrument made from a deer antler and a bivalve shell. However, one of the most celebrated specimens were of human remains, known as the human from Pontimelos or Fontezuelas (Bond, 1999; Hansen, 2019, 2020; Sánchez-Villagra et al., 2023; Weigelt, 1951). Well-preserved and roughly complete although disarticulated, this human skeleton was covered by a fragment of a carapace of *Glyptodon* sp. The finding was extensively debated at that time, because according to some scientists including Roth, it supported the contemporaneity of humans and megafauna in South America. Despite this hypothesis was later proven to be correct, further studies in the man from Fontezuelas revealed that the association was accidental and that carbon dating (14C) found the human remains aged approximately to 1985 ± 15 years BP (Politis & Bonomo, 2011).

In 1885, Lausen purchased another collection of fossils from Roth: the complete Catalogue N° 3 with 194 specimens (Hansen, 2019). The shipping of these fossils from Catalogues N° 2 and N° 3 to Denmark took more than 2 years (1885–1888) and seven separate shipments (Hansen, 2019).

Other Roth collections have a more obscure history. According to Machon (1925, see also Weigelt, 1951, who we follow), the catalogue published by Carolus F. Hofer & Soc. In 1879 corresponds to the first from Roth. However, Roth’s name does not appear in the text. Hofer was his brother-in-law living in Genova (Weigelt, 1951) and acted as his sale partner, at least in some cases. For example, the 1888 Catalogue N° 5 has the following notice (Roth, 1888b): “*Pour traiter on est prié de s’adresser à Santiago Roth è, Kiisnacht, Zurich, ou aussi à Carlo F. Hofer & Co. à gênes, Italie.*”

Catalogue N° 1 is written in Latin and comprises 63 specimens. The geographic origin of the fossils is not provided, although the taxonomic representation agrees with fossils from the Quaternary of the Pampean region. According to Weigelt (1951), Roth shipped the collection from Argentina to Europe to be inspected by the medical doctor and naturalist Prof. August Christoph Carl Vogt who became interested in buying the fossils for the museum in Geneva. The fossils arrived broken in pieces and consequently Roth travelled to Europe in 1880 to personally restore the material. For this task, he also received the assistance of his brother Hermann Roth, who by then lived in Paris. The work was successful and a public subscription was decided to collect funds for the purchase (Weigelt, 1951); however, the money finally offered by Geneva was not enough and other alternative acquirers were needed. Following Carlini et al. (2016), Roth sold fossils in Switzerland in 1880. Today, a collection of Pampean fossils from Roth are housed at the Muséum d’histoire Naturelle de Genève—MHNG and include more than 100 specimens (JDCB, personal observation). It is possible that these fossils correspond to, or are a part of, Catalogue N° 1. Alternatively (or in addition), they may correspond to uncatalogued fossils collected by Roth sold independently to his more elaborated inventories (such as the list of eight fossil materials presented by A. Dreyer to Geneva in 1893; see Sánchez-Villagra et al., 2023). Unfortunately, the MHNG does not record any associated catalogue to the Roth’s material housed in the institution (L. Cavin, 2023 pers. comm. to J.D. Carrillo-Briceño). Another possibility is that specimens from Catalogue N° 1 were acquired by Lausen and donated to ZMK (Ruiz-Ramoni et al., 2023).

Fossils in Roth Catalogues N° 4 to 6 were sold to Swiss Museums (Hansen, 2019). We were able to trace a clear history only for Roth Catalogue N° 5 (see below). In contrast, we were unable to find any information about Catalogue N° 4 (see also Machon, 1925). Catalogue N° 6 comprises 136 numbered specimens and was published in 1892 after Roth’s departure from Europe to Argentina. The collection stayed in Europe under the care of his wife, Elisabeth Schütz (Summermatter, 2012; Weigelt, 1951) who very much helped in his work along his life (Weigelt, 1951) and in that opportunity may have sold the fossils in 1892 or thereafter. The final destination of this collection is unclear.

In Switzerland also the Musée Cantonal de Géologie Lausanne—MCGL houses several fossils from the Pampean region collected by Roth, few were donated by George Claraz. However, there is no record if these fossils were part of any of Roth’s catalogues. In addition, museums at London and Paris also bought some fossils from Roth, but isolated specimens not complete collections (Weigelt, 1951).

In 1895 Roth was incorporated as staff member of the MLP. In September that year, the MLP incorporated a fossil vertebrate collection gathered by Roth in Buenos Aires Province, consisting in 183 mammal specimens (MLP Record Book N°1, Folios 1–16). The stratigraphic and geographic origin of these fossils are “… *depósito de loess fluvio terrestre, formación pampeana intermediar [Mittlere pampasformation (*= *pampeano intermediar or intermédiate pampean)], barrancas del Paraná, Baradero.*” All these fossils were labeled with the letter “P. ” (= “pampean formation”). Probably Roth sold this collection to the MLP before his contract and possible these fossils correspond to, or are part of, some of the catalogues whose final destination were museums of Europe.

## The Roth collection at Zurich: Catalogue N° 5

**Fig. 7:**
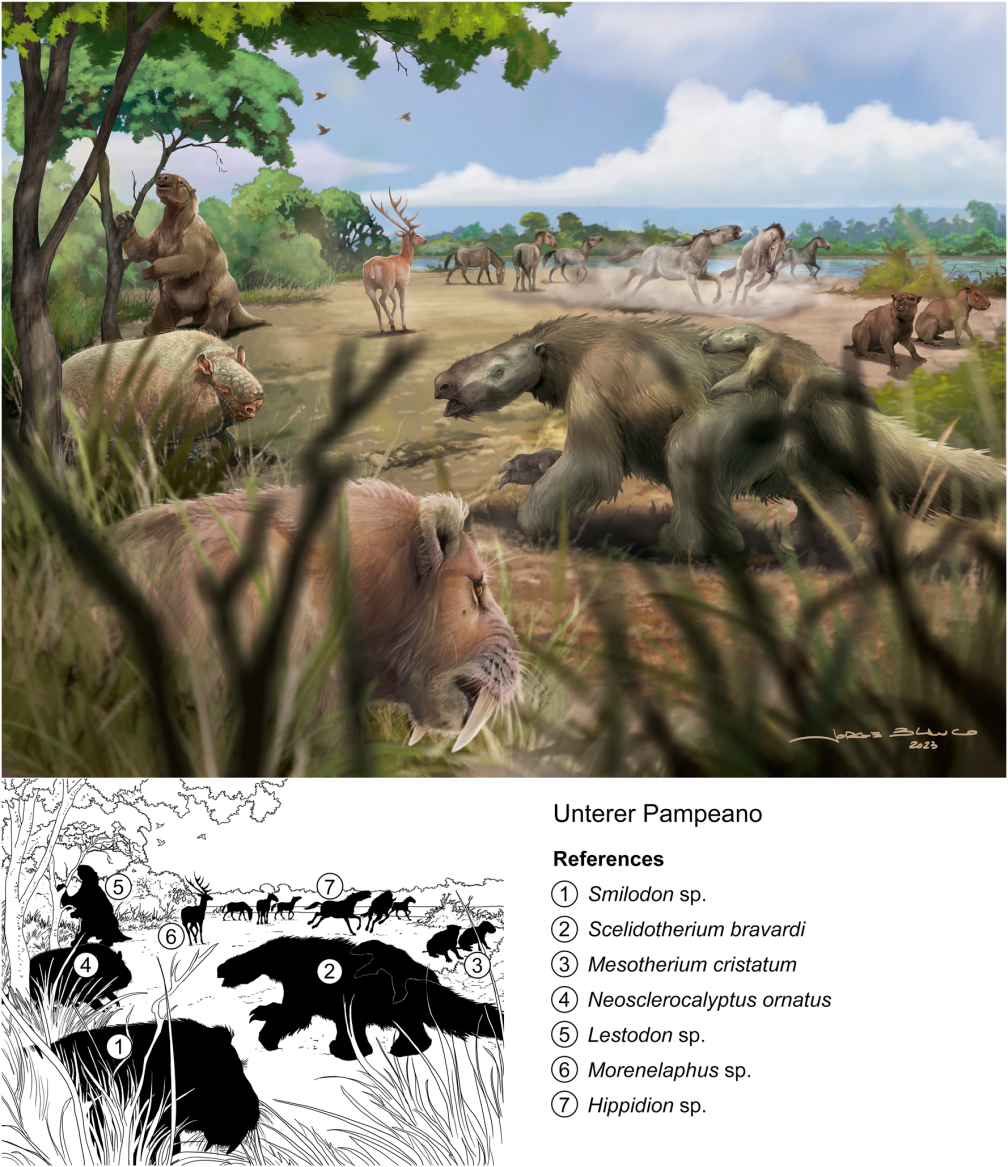
Unterer Pampeano. Landscape of Pampean region at the Early Pleistocene. Roth´s Unterer Pampeano partially correlates with the Ensenadan Stage/Age. The reconstruction is based from specimens from Roth Catalogue N° 5 and MHNG. (**1**) *Smilodon* sp. (Felidae), likely MHNG GEPI V-3213, 3214 (Pleistocene in collection catalogue, exact stratigraphic location uncertain; Ruiz-Ramoni et al., 2023); the presence of this taxon in South America dates from the Early Pleistocene, Ensenadan Stage/Age to the Late Pleistocene, Lujanian Stage/Age (e.g., Prevosti & Forasiepi, 2018); (**2**) *Scelidotherium bravardi* (Mylodontidae), PIMUZ A/V 506, 507, 519, 520 (Le Verger, 2023); (**3**) *Mesotherium cristatum* (Mesotheriidae), PIMUZ 467 (Carrillo & Püschel, 2023); (**4**) *Neosclerocalyptus ornatus* (Glyptodontidae), PIMUZ A/V 447 (Le Verger, 2023); (**5**) *Lestodon* sp. (Mylodontidae), PIMUZ A/V 503 (Le Verger, 2023); (**6**) *Morenelaphus* sp. (Cervidae), PIMUZ A/V 4162 (Carrillo-Briceño et al., 2023); (**7**) *Hippidion* sp. (Equidae), PIMUZ A/V 4240 (Carrillo-Briceño et al., 2023). Reconstruction by Jorge L. Blanco

**Fig. 8:**
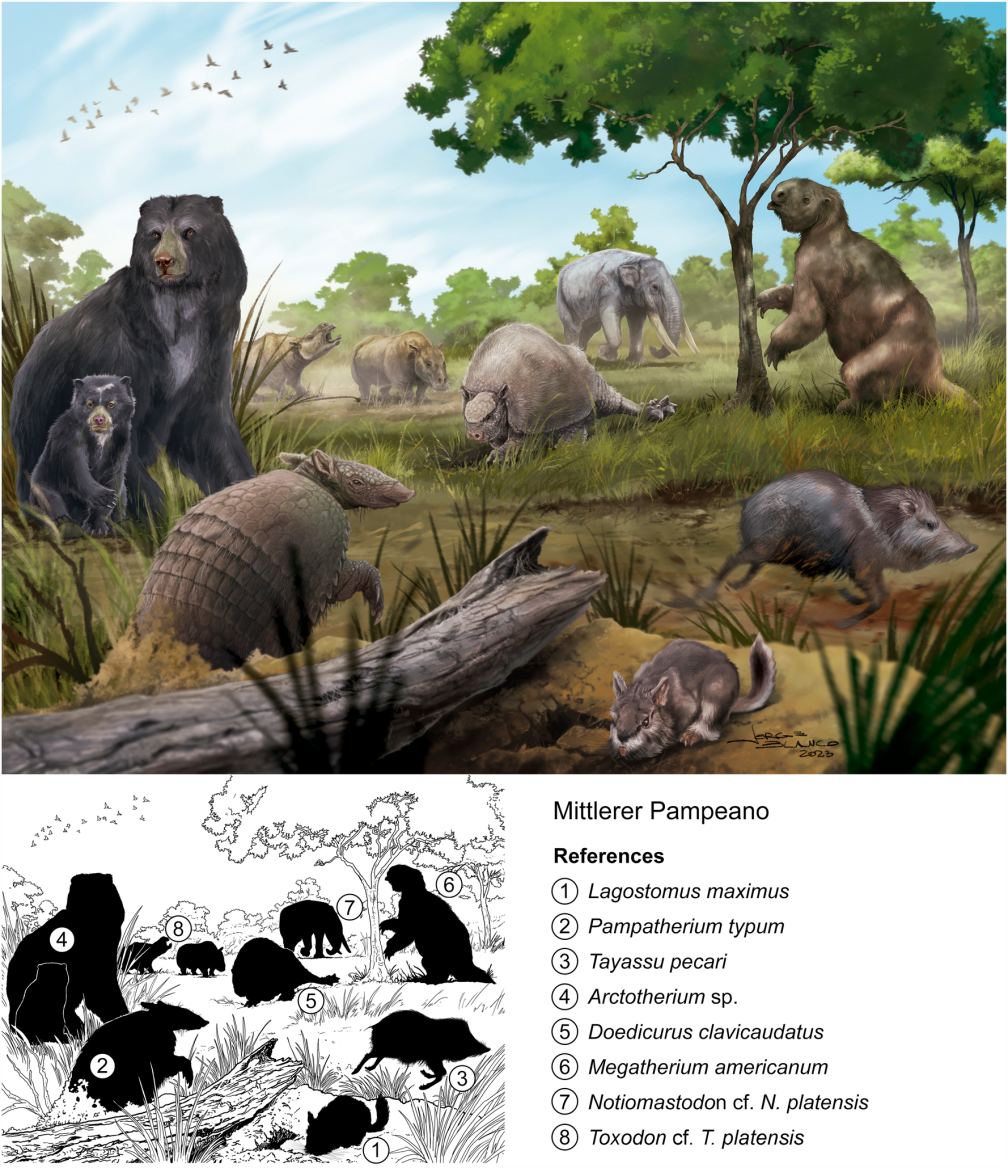
Mittlerer Pampeano. Landscape of Pampean region at the late Early Pleistocene to Middle Pleistocene. Roth´s Mittlerer Pampeano partially correlates with the Ensenadan and likely Bonaerian Stage/Age. The reconstruction is based from specimens from Roth Catalogue N° 5. (**1**) *Lagostomus maximus* (Chinchillidae), PIMUZ A/V 4235a, 4235b, 4202 (Kerber, 2023); (**2**) *Pampatherium typum* (Pampatheriidae), PIMUZ A/V 431, 432 (Le Verger, 2023); (**3**) *Tayassu pecari* (Tayassuidae), PIMUZ A/V 4188 (Carrillo-Briceño et al., 2023); (**4**) *Arctotherium* sp. (Ursidae), PIMUZ A/V 4215 (Ruiz-Ramoni et al., 2023); (**5**) *Doedicurus clavicaudatus* PIMUZ A/V 459, 4148 (Le Verger, 2023); (**6**) *Megatherium americanum* (Megatheriidae), PIMUZ A/V 479, 481, 482, 483 (Le Verger, 2023); (**7**) *Notiomastodon* cf. *N. platensis* (Gomphotheriidae), PIMUZ A/V 4161, 4092 (Carrillo-Briceño et al., 2023); (**8**) *Toxodon* cf. *T. platensis* (Toxodontidae) PIMUZ A/V 4163, 4199, 4210, 4233, 4245, 4290, 5697 (Carrillo & Püschel, 2023). Reconstruction by Jorge L. Blanco

**Fig. 9:**
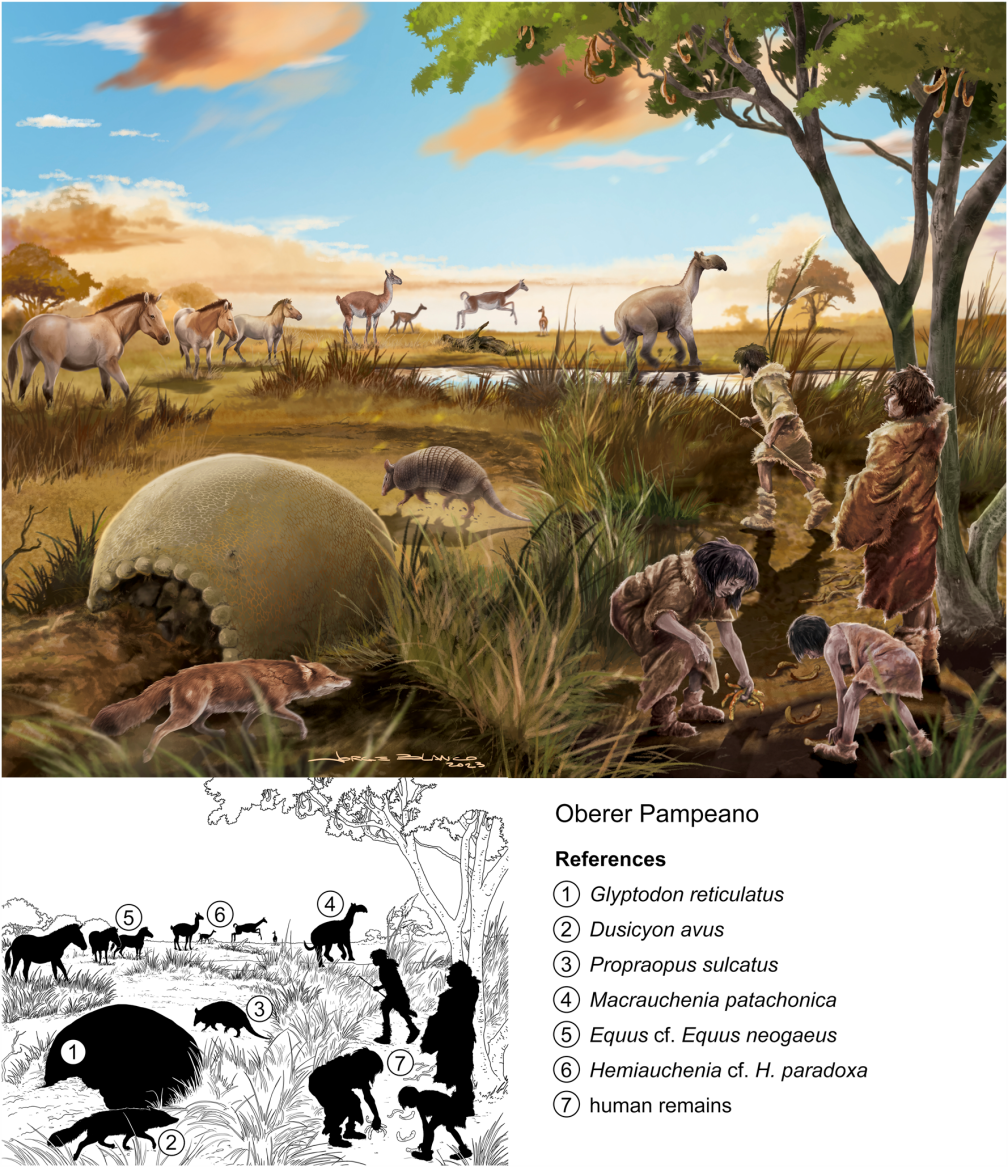
Oberer Pampeano. Landscape of Pampean region at the Late Pleistocene. Roth´s Oberer Pampeano correlates with the Lujanian Stage/Age. The reconstruction is based from specimens from Roth Catalogue N° 5. (**1**) *Glyptodon reticulatus* (Glyptodontidae) PIMUZ A/V 4122 (Le Verger, 2023); *Dusicyon avus* (Canidae), PIMUZ A/V 4232 (Ruiz-Ramoni et al., 2023); (**3**) *Propraopus sulcatus* (Dasypodidae), PIMUZ A/V 426, 427 (Le Verger, 2023); (**4**) *Macrauchenia patachonica* (Macrauchenidae), this species is an artistic license, since all specimens from Catalogue N°5 assigned to this taxon are from the Mittlerer Pampeano (PIMUZ A/V 4118, 4119, 5700; Carrillo & Püschel, 2023; Püschel & Martinelli, 2023); (**5**) *Equus* cf. *Equus neogaeus* (Equidae), PIMUZ A/V 4212, 4248 (Carrillo-Briceño et al., 2023); (**6**) *Hemiauchenia cf. H. paradoxa* (Camelidae), PIMUZ A/V 4186, 4195, 4127, 4196, 4255) (Carrillo-Briceño et al., 2023); (**7**) Findings of humans remains in some archeological sites of Buenos Aires Province, such as Fontezuelas or Baradero, influenced Roth´s interpretation on the contemporaneity with megamammals in the "pampean formation" (Sánchez-Villagra et al., 2023). Reconstruction by Jorge L. Blanco

On the recommendation of geologist Albert Heim and the zoologist Arnold Lang, the Federal Council of Switzerland, the canton of Zurich, and private donators purchased in 1890 the Roth collection from Catalogue N° 5. This collection is composed mainly of mammals including nicely preserved skeletons, a few turtle carapace fragments, a fragment of silicified wood, and a few fish teeth from the Pampean region of Argentina (Figs. 7, 8, 9). Catalogue N° 5 includes a total of 284 catalogued specimens (Additional file 1). However, two are unnumbered specimens: one is an inconsistency and the other adds one more specimen to the list. The first unnumbered specimen is a hindlimb of *Scelidotherium leptocephalum,* indicated below specimen number 65 on the list, but it may belong to the same specimen listed as number 52 (PIMUZ A/V 510) (the specimen was split, likely an editing error). The second unnumbered specimen relates to number listed as 277 (PIMUZ A/V 514), which includes two different elements: a vertebra from Río Carcarañá and a partial zygomatic arch from Arroyo del Medio, both assigned to Scelidotherinae indet (Le Verger, 2023). Both specimens were labeled with the same catalogue number (likely the result of a printing error).

A copy of the Catalogue N° 5 from Roth (1889) at Zurich containing handwritten numbers, probably indicated suggested prices in Swiss francs (Additional file 1). For example, the first listed specimen (PIMUZ A/V 506) assigned to the ground sloth, *Scelidotherium bravardi,* includes a nicely preserved partial cranium, dentary, five ribs, one cervical vertebra, fragment of vertebral apophysis, right femur and pes, left scapula, femoral head, tibia, and pelvic fragment (Le Verger, 2023), and has the associated handwritten number “1299”. Specimen number 146 (PIMUZ A/V 4240), referred to *Hippidion* sp., is an isolated m3 (Carrillo-Briceño et al., 2023) and is associated with the number “2” (Additional file 1). The sum of these handwritten numbers 67,309, what could mean a total price of about 70,000 Swiss francs. At current estimated value, the collection cost was over EUR 350,000.

At the time, an “appeal to the public” (see Heim & Lang, 1890) was printed with a short description of the unique collection of 284 catalogued fossils from the “Pampasformation” of Argentina, from the catalogue published by Roth (1889). This document underlined the scientific value of the collection and indicated a financial cost of more than 80,000 Swiss francs. Roth asked for only 40,000 Swiss francs. When Switzerland purchased Roth Collection No. 5, Albert Heim signed as director of the geological collections at the “Polytechnikum Zürich” (today the Swiss Federal Institute of Technology in Zurich, ETH from Eidgenössische Technische Hochschule Zürich in German) at the Polytechnikum and University of Zurich (UZH), as did Arnold Lang, as director of the zoological collections at the “Polytechnikum” and professor of zoology at the Polytechnikum and University of Zurich.

In 1893, Heim and Lang thanked the local Swiss community, several organizations and societies, the government of Canton Zurich, and the Federal Council of Switzerland for their contribution and invited them for a visit to the central hall of the Swiss Federal Institute of Technology in Zurich (Heim & Lang, 1893). They raised a total of Fr. 50,748.70 which was mainly used to pay for the Roth collection (Fr. 40,000) and for fossil preparation by a technician (A. Dreyer) during more than 18 months (Fr. 8550.15). The remaining amount of Fr. 1791.25 was used for the preparation of mammoth fossils from Niederweningen (Canton Zurich), discovered in 1890 and also exhibited in the “Polytechnikum Zürich”. Approximately Fr. 407.30 remained for the zoological collection.

The “Santiago Roth’sche Sammlung” was exposed in a large glass display from 1893 to 1909 as part of the Palaeontological collection in the main building of the “Polytechnikum Zürich”. Roth himself never saw this exhibition. In 1891, after the purchase of fossils was complete, he was already back in Argentina and never returned to Switzerland (Hansen, 2019; Weigelt, 1951). For his merits as a collector and researcher, in 1900 the University of Zurich honored Santiago Roth with the title *Philosophiae Doctor, honoris causa*.

Since 1909, the Roth Collection is property of Canton Zurich alone, after an agreement between the Federal Council of Switzerland and the Government of the Council of Zurich. In 1914, all the fossils were transferred to the new Zoological Museum in the new building of the University of Zurich.

In 1919, Betty Schulthess (Zurich) finished her PhD. thesis as one of the first female students at the University of Zurich, reviewing all of the Roth collection material. A year later she published (Schulthess, 1920) her detailed morphological descriptions and systematic determination, and analyzed in particular elements of manus and pes of xenarthrans.

Since 1956, all the fossils were curated by the newly established Palaeontological Institute of the University of Zurich under the direction of Emil Kuhn–Schnyder. Later, the exhibition was drastically reduced by the Zoological Museum and most fossils of the Roth collection were stored in repositories of the PIMUZ. Original numbers in Roth’s Catalogue N° 5 (Roth, 1889) were supplemented by new inventory numbers (PIMUZ A/V), using locality information from Roth (1889). All information is available in the electronic database: https://www.pim.uzh.ch/apps/cms/pageframes/sammlung_db.php.

Since the renovation of the Zoological and Palaeontological museums in 1991, only two newly mounted skeletons, and partially supplemented skeletons of *Megatherium americanum* PIMUZ A/V 479 and *Glyptodon munizi* PIMUZ A/V 461 are exhibited in the zoological part of the Museum (Le Verger, 2023: Fig. 1). These spectacular reconstructions were also the main objects in a temporary exhibition.

## Preservation of the Roth collection at the Department of Paleontology, University of Zurich

The “Roth Sammlung” (Roth collection) from Catalogue N° 5 originally included a total of 284 specimens (Roth, 1889; Figs. 7, 8, 9). It is preserved almost in its entirety at PIMUZ with most specimens being fossil mammals (98.5%), while turtles (1%) and fishes (0.5%) are minority groups. Among mammals, the majority is comprised of xenarthrans (Le Verger, 2023; Schulthess, 1920) followed by Holarctic ungulates (Carrillo-Briceño et al., 2023).

A preservation treatment is currently underway to ensure the collection’s long-term stability and to promote future research. We describe the treatment here, highlighting critical conservation steps taken to protect the collection’s physical integrity and its associated field and catalogue data.

### Pre-treatment conditions

**Fig. 10:**
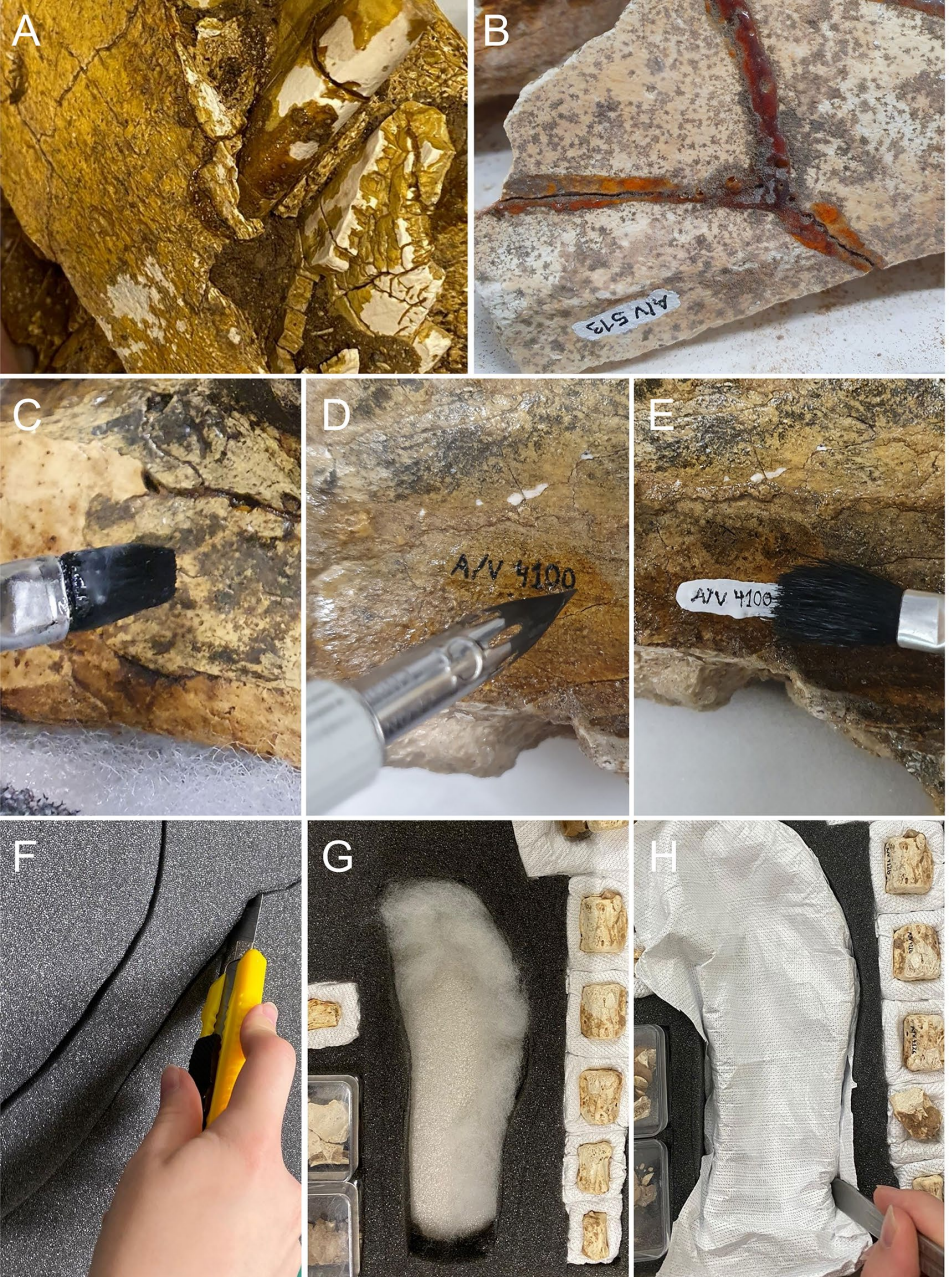
Preservation treatment on Roth´s collection Catalogue No. 5 at PIMUZ. Unidentified, old consolidant ageing: yellowing and flaking (**A**). Unidentified adhesive, yellowed, sticky, and chemically irreversible (**B**). Labelling in 3 steps: application of 20% Paraloid B-72 in acetone as a protective base layer (**C**); label written with india ink and quill (**D**), and gentle application of 20% Paraloid B-72 as a top coat (**E**). Application of the top coat may sometimes cause numbers to run. Sufficient drying time is needed between each layer. A 50% Paraloid B-72 solution was sometimes used as a top coat. In E, the label was written on a layer of titanium white acrylic paint, after the base coat, for contrast. Housing process: "Cavity" mount out of ethafoam according to specimen’s contours (**F**); cushion the cavity with polyester batting (**G**), and cut outline around cavity and tuck Tyvek material (42 g/m^2^) into a slit to secure in place (**H**) (Dzinak, 2017)

The original treatment of the fossils was carried out by the technician A. Dreyer during 1890–1893, instructed at the beginning by Roth himself. Since the arrival at PIMUZ, Roth Collection No. 5 had not been treated with modern conservation techniques and practices. Consequently, adhesives and consolidants had begun to fail, specimen labels were deteriorated, and some specimens themselves were in disrepair. Most of the material had been heavily coated with a varnish-like substance, likely as a form of consolidant, which has now darkened and flaked (Fig. 10). Many specimens were not stored as to prevent their damage in storage or during handling, or for the retention of association between elements. Examples include friable bone elements dispersed among heavier ones, lack of friction deterrents, separation of associated specimens, and general deterioration due to time. Most importantly, specimen data were at risk for loss due to non-archival labelling. Finally, some material had never been curated.

### Conservation treatment

Based on these conditions, a conservation treatment was designed to (1) remove old coatings/materials that failed over time, (2) consolidate and repair fractured, fragile specimens with conservation-grade materials, (3) apply archival labelling and reduce data loss, and (4) re-house the collection. Table 1 lists some of the specimens that were treated first, due to their fragile condition and relevance to ongoing studies.

**Table 1:** Sample of specimens from the Roth Collection at the Paleontological Institute and Museum, Zurich treated for preservation with new curatorial techniques

Specimen ID	Taxon	Element(s)
PIMUZ A/V 4126	*Eutatus seguini*	Vertebra and a part of a rib
PIMUZ A/V 467	*Mesotherium cristatum*	Skull and lower jaw fragments
PIMUZ A/V 4283	Mammalia indet	Possible radius
PIMUZ A/V 4132	Equidae indet	Vertebra
PIMUZ A/V 4100	*Hippidion* cf. *H. principale*	Upper jaw fragments
PIMUZ A/V 4149	*Scelidotherium leptocephalum*	Skull and jaw fragments
PIMUZ A/V 4164	*Mammalia* gen. et sp. indet	Posterior limb and patella
PIMUZ A/V 513	*Scelidotherium leptocephalum*	Probably ribs, clavicle, patella, and others

1. Cleaning/removal of old coatings: Specimens were inspected for structural stability. When stable, small areas were tested for reversibility of surface coatings using organic solvents. Most coatings were irreversible, likely having cross-linked over time. For some specimens, i.e., PIMUZ A/V 513 (see Table 1), coatings became tacky and were partially reversible after multiple rounds of cleaning with acetone. Results revealed previously-covered morphology. In some cases, treatment exposed areas that were artificially reconstructed which could otherwise be misinterpreted as real.
2. Repair and consolidation: Paraloid B-72, also known as Acryloid B-72 (Paraloid/Acryloid B-72 is a resin made by Rohm and Haas, USA), is an acrylic copolymer composed of methyl methacrylate and ethyl acrylate, used in the conservation of archaeological and paleontological materials (Beaubien, 2019; Davidson & Goldberg, 2014; Koob, 1984, 1986). As a class “A” solution adhesive/consolidant (Horie, 2010), it is known for exceptional aging properties: long-term reversibility, clarity, lack of chemical cross-linking, resistance to light, and theormoplasticity (Beaubien, 2019; Davidson & Goldberg, 2014; Koob, 1986). Paraloid B-72 is soluble in acetone, ethanol, or a combination of both organic solvents. Mixed in different concentrations it can be adjusted for different purposes or substrates. Solutions in ethanol will set more slowly and thus potentially increase the likelihood of substrate penetration. Tests on small areas are always recommended before full treatment.
  A solution of 5–10% Paraloid B-72 in acetone was used for most consolidation. After cleaning, it was generously applied to the most fragile specimens, i.e., PIMUZ A/V 416, and allowed to penetrate for at least 1 h. Treatment was repeated as needed until specimens were considered safe for manual handling. Note: when this type of consolidant is too concentrated, it may leave a shiny top coat on the substrate, but is reversible with additional acetone. For our treatment, concentrations ranged between 5 and 15% Paraloid B-72 in acetone, depending on specimen.
  Repairs were made with a solution of 50% Paraloid B-72 in acetone—a mixture that is viscous and dries slowly, and has an estimated working time of approximately 10–15 min. Broken surfaces were first primed with a consolidating solution of 5–10% Paraloid B-72 in acetone to improve the strength and uniformity of the bond (Koob, 1986). This is most important for extremely porous surfaces. More than one priming coat was necessary in most cases. The adhesive (50% Paraloid B-72 in acetone) was then applied to both fragments before assembly, and allowed to set for several days or weeks. During this time, uniform pressure was maintained with sand bags, to ensure the best possible join. Repairs of this type can look “set” very quickly, but avoiding handling for at least one day is recommended. Full hardness (strength) of Paraloid B-72 adhesive can take weeks or months to be fully achieved. As an adhesive, it is reversible with acetone.
3. Labelling and data protection: Labels written directly on specimen surfaces began with a base coat of 20% Paraloid B-72 in acetone. Such labels were applied only to areas that were not diagnostic or scientifically-important, i.e., avoiding teeth and sutures. For dark-toned specimens, a layer of titanium white acrylic paint (diluted with water) was applied atop the base layer to contrast with dark ink. Numbers and other data were written with carbon-rich ink (“india ink”) using a quill, or with Pigma® Micron pens (Pigma is a registered trademark of the Sakura company). A top coat of 20% Paraloid B-72 in acetone sealed the labels (Fig. 10). Lastly, old and new paper labels were placed into polyethylene sleeves for protection from handling, moisture, and wear.
4. Re-housing: Most specimens were rehoused in acid-free cardboard boxes to ensure long-term safety in storage (European vendors be provided upon request). High-priority specimens were housed in cavity mounts (housings with cushioned depressions shaped to the specimen) which were made with the following archival materials: (a) ethafoam sheeting (2 mm) (polyethylene, PE) and planks (20 mm), (b) polyester batting, and (c) Tyvek® polyethylene sheeting weighing 41 g per square meter (Tyvek is a registered trademark of the Dupont company). Ethafoam is a common packaging material that is often discarded. Part of the supply for this treatment came from recycled material. Tyvek® is a waterproof, protective material known for its strength and durability. Cavity mounts were made as follows (Fig. 10) (Dzinak, 2017):
  4.1 Cavity: ethafoam sheeting was cut to line the bottoms of acid-free paper trays and boxes. The fossil’s shape was traced onto thick ethafoam planks (same dimensions as the box), cut and removed, leaving a cavity in the shape of the specimen.
  4.2 Cushion: the cavity was cushioned with polyester batting, creating a shock-absorbing bed for the specimen.
  4.3 Lining: an incision was made into the ethafoam plank ~ 1 cm away from the cavity’s edge. Tyvek covering the cavity was tucking and secured into this incision. For more information see https://nhm.org/person/zdinak-alan.

### Structural supports

Specimen PIMUZ A/V 416 was assessed as a high-risk specimen: a tibia and fibula with a metal screw drilled through both elements to hold them together. Bone areas in contact with the screw were heavily damaged. Ethafoam padding was nestled between the metal and fossil, and weight-bearing points were chosen during re-housing to reduce gravitational stresses on weaker areas. Severely damaged areas were stabilized with adhesive-strength Paraloid B-72 in acetone, to prevent torsion.

### Sustainability

Some of the ethafoam used in this pilot study was salvaged from packing materials designated as waste. Only materials made of polyethylene (PE) were used, as it is known to be inert (no off-gassing), unlike other commercial “foams”. Packing “peanuts”, for example, are often made from materials that are not necessarily inert. Tyvek® for this project was obtained from a local industrial provider that was disposing it. Local industrial suppliers may welcome takers of their “scrap” polyethylene materials as a way to reduce waste.

### Results and future perspectives

Conservation of natural history collections is a time-intensive process but is crucial to the long-term preservation of fossil specimens and data. Treatments described here are in line with recommendations of other natural history institutions in Switzerland and elsewhere (Frick & Greef, 2021) and are meant as guidelines that can be adjusted to the needs of individual collections. Efforts to ensure the safety and longevity of the PIMUZ Roth collection continue. Current plans include moving this valuable collection to a new climate-controlled collection center for university cultural assets (Universität Zürich Zentraldepot) in Buchs, Zurich, with adequate space and research access.

## Digitalization

Twenty-three skeletal elements from the Roth collection, preserved at PIMUZ, were scanned using the Artec Space Spider and Eva structured blue light scanners (Table 2).

**Table 2:** Scanned specimens from the Roth Collection at the Paleontological Institute and Museum, Zurich available at [https://sketchfab.com/PIMUZ]

Specimen ID	Taxon	Element(s)
PIMUZ A/V 419	*Eutatus pascuali*	Skull
PIMUZ A/V 438	*Neosclerocalyptus paskoensis*	Incomplete skeleton
PIMUZ A/V 439	*Neosclerocalyptus pseudornatus*	Skull
PIMUZ A/V 450	*Neosclerocalyptus *sp*.*	Tail
PIMUZ A/V 463	*Glyptodon munizi*	Bone
PIMUZ A/V 465	*Glyptodon munizi*	Incomplete skeleton
PIMUZ A/V 471	*Glyptodon munizi*	Incomplete skeleton
PIMUZ A/V 477	*Nothrotherium escrivanense*	Incomplete skeleton
PIMUZ A/V 484	*Glossotherium robustum*	Incomplete skeleton
PIMUZ A/V 491	*Lestodon armatus*	Skull
PIMUZ A/V 493	*Lestodon armatus*	Lower jaw
PIMUZ A/V 509	*Scelidotherium leptocephalum*	Incomplete skeleton
PIMUZ A/V 510	*Scelidotherium leptocephalum*	Skull
PIMUZ A/V 511	*Catonyx tarijensis*	Skull
PIMUZ A/V 512	*Catonyx tarijensis*	Skull
PIMUZ A/V 513	*Scelidotherium leptocephalum*	Incomplete skeleton
PIMUZ A/V 4216	*Macrauchenia patachonica*	Incomplete skeleton
PIMUZ A/V 5700	*Macrauchenia patachonica*	Incomplete skeleton

Medium-size samples (smaller than 180 × 140 mm) were acquired using the Artec Space Spider, whereas large-size specimens (larger than 214 × 148 mm) were digitized using the Eva. The scanners used can achieve sub-millimetric 3D resolution in the final model up to 0.1 mm and 0.2 mm and point accuracy of 0.05 mm and 0.1 mm (Space Spider and Eva, respectively). Each specimen was placed on a rotatory platform and scanned in different positions to register its geometry and texture. This process resulted in up to four scans per specimen. When using the Artec Space Spider, each capture was made at a distance of about 20–30 cm. The working length for collections acquired using Eva was 0.4–1 m. The time of capture for each specimen was between 10 and 15 min.

**Fig. 11:**
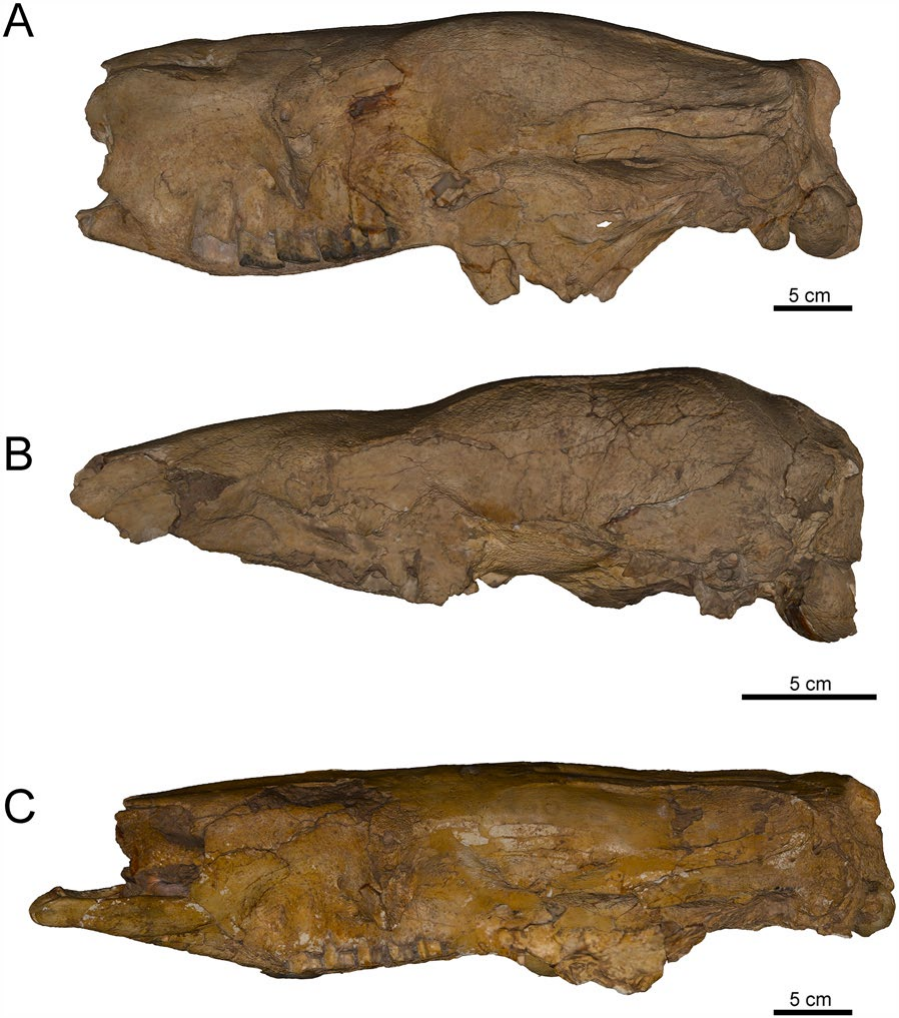
Specimens scanned using the Artec Space Spider and Eva structured blue light scanner. The detail surface and color are captures in great detail. *Catonyx tarijensis* PIMUZ AV 512 (**A**); *Nothrotherium escrivanense* PIMUZ AV 477 (**B**); *Scelidotherium leptocephalum* PIMUZ AV 513 (**C**). Specimens available at [https://sketchfab.com/PIMUZ]

The raw scan data were processed using Artec Studio 17 Professional. First, a *Fine registration* was performed to align the sequential frame pairs on the scans captured on each specimen. Second, the *Auto-alignment tool* matched the overlapped scans in the same 3D space. Third, a *Global registration* was completed to compare and optimize the frame position across all scans. Fourth, small outlier surfaces unconnected to the main mesh were deleted to clean up the edge noise in the final model. Five, the *Sharp Fusion* tool was used to fuse all the scans and create a single high-resolution mesh. Finally, interpolation and normalization of textures were applied to the models to obtain a realistic appearance. Texture parameters such as brightness, gamma correction, and contrast were adjusted to resemble the original specimen. The resulting high-quality meshes were exported in .stl (i.e., mesh) and .obj (i.e., mesh and texture) formats (Fig. 11).

As previously done in small and large museums worldwide (Erolin et al., 2017; Rangel-de Lázaro et al., 2021; Younan & Treadaway, 2015; see also Kerber, 2023; Püschel & Martinelli, 2023), the 3D scanners used proved to be a fast method that allowed us to reach adequate results in detail and accuracy. The acquisition and post-processing methodology followed allowed us to create a reality-based 3D data set reproducing the geometry and textures of the original specimens. The 3D models produced can be examined now using free 3D viewers and modelling software. As we advance, the 3D models will be upload into Sketchfab to increase the visibility of the Roth collection. The online presence of this virtual collection may significantly increase the visibility and value of the specimens safeguarded by the institution.

## Concluding remarks

Santiago Roth (Herisau, Switzerland, 14th June 1850—Buenos Aires, Argentina 4th August 1924) is a renowned figure in the field of paleontology in Argentina. Housed in Buenos Aires Province, his original investigations provided much of the basis to understand the stratigraphy of Pampean region interacting and sometimes confronting the hypotheses of another celebrated figure of the time, Florentino Ameghino. Roth was a multifaceted person. Results of his work also include large collections of fossils vertebrates, most of them megamammals, and in a lesser extent archaeological remains from the Quaternary of Pampean region. Some of these specimens triggered international debates, such as the contemporaneity of megamammals and humans in South America. In Argentina, his name is connected to the Museo de La Plata, where he worked for around 30 years. In Europe, it is linked to Switzerland (Musée Cantonal de Géologie Lausanne, Muséum d’histoire Naturelle de Genève, Palaeontological Institute and Museum of the University of Zurich) and Denmark (Zoologisk Museum, København), where he sold many precious fossils that are still today inspiring material for new research.

### Supplementary Information

**Additional file 1. **Additional information accompanies this paper at: Roth, S. 1889. Fossiles de la Pampa. Amérique du Sud. Catalogue No. 5. Zürich.

## Data Availability

PIMUZ fossil collection database: https://www.pim.uzh.ch/apps/cms/pageframes/sammlung_db.php 3D models from specimens of the Roth collection at PIMUZ available at Sketchfab: [https://sketchfab.com/PIMUZ].
