# Supplementary material for: Pampean megamammals in Europe: the fossil collections from Santiago Roth
Source: Swiss J Palaeontol. 2023 Sep 29;142(1):25. doi: 10.1186/s13358-023-00283-5 (PMC10542304; doi:10.1186/s13358-023-00283-5)
Supplement: Supplementary file 1 — Additional file 1. Additional information accompanies this paper at: Roth, S. 1889. Fossiles de la Pampa. Amérique du Sud. Catalogue No. 5. Zürich. [file 13358_2023_283_MOESM1_ESM.pdf]

FOSSILES DE LA PAMPA

AMÉRIQUE DU SUD

collectionné par

SANTIAGO ROTH

SAN NICOLAS

RÉPUBLIQUE ARGENTINE

CATALOGUE No. 5

ZURICH
Imprimerie Jean Meyer
1889

Collection No. 5.

1. Restes d'un jeune Scelidotherium Terrain pampéen inférieur. (Arroyo Pergamino). 1200
2. Un crâne sans mâchoire inférieure d'une très grande espèce de Scelidothérium. Pampéen moyen. (Barranca del Parana San Nicolas). 1000
3. Un fragment d'étui caudal, Pamp. moyen. (Arroyo Pergamino). 4
4. Un tibia d'un milodon, Pamp. moyen. (Arroyo del Medio). 10
5. Un fragment de crâne d'un Scelidotherium. Pamp. supérieur. (Barranca del Parana, villa Constitucions. 30
6. 2 fémurs, un péroné et tibia, une patte postérieur, incomplète, une omoplate, un humérus, un radius. un cubitus, une patte anterieure, une vertèbre cervical, un fragment de la carapace d'un Glyptodon. Pamp. moyen. (Arroyo Pergamino). 800
7. Un morceau de bois silicifié (Rio-Negro, Patagonie).
8. Une vertèbre provenant probablement d'une espèce de l'ordre des cétacés des Terrains Entre-riano (Curtiembre). 20
9. Une corne de cerf. Terrain pamp. inf. (Baranca del Parana, San Nicolas). 50
10. Un tibia et quelques os du pied d'un Scelidotherium Pamp. sup. (Baranca del Parana, San Nicolas). 30
11. Quelques os d'un pied. Pamp. moyen. (Barranca del Parana, San Nicolas). 10
12. Quelques vertèbres caudales de Scelidotherium. Pamp. sup. (Pergamino). 30
13. Un fragment de crâne et un os du pied d'un Scelidotherium. Pamp. moyen. (Baranca del Parana, San Lorenzo). 100
14. Un fragment d'un étui caudal. Pamp. moyen. (Barranca del Parana. San Nicolas). 10

15. Un cubitus. Pamp. moyen. (Punta alta Bahia Blanca). 50
16. Une machoire supérieure d'une grande espèce d'Equus. Pamp. moyen. (Rosario). 500
17. Quelques plaques de la carapace d'un Panochtus du Terrain pamp. sup. (Rio Tercero). 20
18. Un crâne avec mâchoire inférieure d'un très petit Scelidotherium. Pamp. moyen. (Barranca del Parana, Villa Constitucion). 200
19. Quelques dents d'un Scelidotherium. Pamp. sup. (Bar. del Parana, Villa Constitucion). 30
20. Un morceau d'une carapace d'Hoplophorus, Pamp. sup. (Rio tercero, Villa Maria). 20
21. Un morceau d'une carapace et un pelvis d'un Glyptodon. Pamp. moyen. (Arroyo dulce). 300
22. Restes d'un Scelidotherium. Pamp. moyen. (Arroyo del Medio). 200
23. Un fémur, un radius, 2 cubitus, quelques os du pied, 2 vertèbres de la queue d'un Lestodon, très fracturés. Pamp. infér. (Barranca del Parana, San Lorenzo). 300
24. Un étui caudal, quelques anneaux de la queue, incomplets; quelques morceaux de la carapace d'un Hoplophorus. Pamp. inf. (Barranca del Parana, Rosario). 400
25. Un crâne d'un Scelidotherium. Pamp. moyen. (Arroyo Pergamino). 300
26. Un morceau de la carapace d'un Glyptodon, Pamp. inf. (Arroyo Pergamino). 200
27. Un morceau de la carapace et de l'écunon de la tête d'un Eutatus. Pamp. moyen. (Arroyo Pergamino). 100
28. Un morceau de la carapace d'un Hoplophorus. Pamp. sup. (Rosario). 50
29. Un pelvis et un morceau de la carapace d'un Glyptodon. Pamp. inf. (Arroyo Pergamino). 200
30. Reste d'un Scelidotherium. Pamp. intermédiaire. (Arroyo del Medio). 200
31. Un pelvis de milodon. Pamp. inf. (Arr. Pergamino). 200
32. Un morceau de la carapace d'un Hoplophorus. Pamp. sup. (Arr. Pergamino). 50
33. Un crâne de Toxodon incomplet. Pamp. intermédiaire. (Arroyo Pergamino). 250

34. Un morceau de la carapace d'un Glyptodon. Pamp. inf. (Arroyo Pergamino). 100
35. Une queue de Glyptodon, incomplète. Pamp. sup. (Baradero). 300
36. Une mâchoire inférieure de Toxodon. Pamp. intermédiaire. (Arroyo Pergamino). 250
37. Un tibia, trois phalanges et dents d'un Scelidotherium. Pamp. sup. (Baradero). 150
38. Un crâne de Palaeoloma. Pamp. inf. (San Lorenzo). 150
39. Os du pied d'un Hoplophorus. Pamp. sup. (San Nicolas.) 2
40. Une mâchoire inférieure de Tipotherium. Pamp. inf.. (San Nicolas). 50
41. Un crâne avec mâchoire inférieure incomplet de Scelidoth. Pamp. sup. (Baradero). 500
42. Un fragment de Fémur, un tibia, une patte postérieure, un cubitus, un radius, une patte antérieure, un morceau de carapace de Glyptodon, Pamp. intermédiaire. (Arroyo del Medio). 500
43. Un morceau de carapace d'un Hoplophorus. Pamp. intermédiaire. (Arroyo del Medio). 20
44. Un crâne avec mâchoire inférieure et quelques os du pied d'un Auchenio ou Palaeolama, Pamp. intermédiaire. (Arroyo Pavon). 200
45. Une moitié de mâchoire inférieure et quelqes os du pied d'un Ursus. Pamp. intermédiaire. (Arroyo Pergamino). 100
46. Un crâne avec mâchoire inférieure incomplet de Palaeolama. Pamp. intermédiaire. (Arroyo Pavon). 300
47. Un crâne de Lestodon. Pamp. intermédiaire. (Villa Constitucion). 500
48. Un tibia de Mastodon. Pamp. sup. (Arroyo Pavon). 20
49. Un morceau de l'étui caudal d'un Hoplophorus. Pamp. sup. (Pergamino). 20
50. Une colonne vertébrale d'une espèce de Biloricata. Pamp. interméd. (Arroyo del Medio). 50
51. Un morceau de la carapace et restes d'un squelette de Panochtus. Pamp. intermed. (Rio Carcaranna.) 500
52. Un crâne de Scelidotherium. Pamp. intermédiaire. (Arroyo del Medio). 200
53. Un fémur d'Eutatus. Pamp. intermédiaire. (Arroyo del Medio). 5

54. Un morceau de la mâchoire inférieure d'un Scelidotherium. Pamp. interméd. (Arroyo del Medio.) 20
55. Articulations du genou d'un Palaeolama. (Pamp. sup. (Pergamino). 5
56. Une incisive de Toxodon. Pamp. interméd. (Arroyo Pergamino). 5
57. Une phalange d'Equus. Pamp. interméd. (Arr. dulce). 2
58. Une mâchoire inférieure d'Eutatus. Pamp. sup. (Pergamino). 10
59. Un fragment de dent d'une petite espèce de Megatherium. Pamp. intermed. (Pergamino). 5
60. Quelques fragments de mâchoire de ruminants. Pamp. sup. (Pergamino). 10
61. Un fragment de mâchoire inférieure de Mastodon. Pamp. interméd. (Arroyo dulce). 150
62. Un radius de Toxodon. Pamp. interméd. (Arroyo Merciel). 100
63. Une molaire de Mastodon. Pamp. intermed. (Arroyo Pergamino). 50
64. Un tibia de Milodon. Pamp. interméd. (Lacuna Barancosa). 20
65. Une molaire de Mastodon. Pamp. interméd. (Arroyo del Medio). 10

Une patte postérieure de Scelidotherium, qui appartient au crâne No. 52.

66. Une surface articulaire d'un humérus de Mastodon. Pamp. interméd.. (Arroyo del Medio). 10
67. Un pelvis d'une espèce de Biloricata. Une plaque de carapace que j'ai trouvée avec le pelvis montre les mêmes particularités que celles du Glyptodon; mais le pelvis diffère très sensiblement de toutes les espèces connues de Glyptodon. Pamp. interméd. (Arroyo dulce). 500
68. Un pied de Milodon. Pamp interméd. (Arr. del Medio. 50
69. Deux fragments de la carapace d'un Panochtus; Pamp. interméd. (Arroyo del Medio). 10
70. Un os de pied. Terrain pamp. interméd. (Arroyo del Medio). 5
71. Une dent de Toxodon; Pamp. moyen (Arroyo Pavon). 5
72. Une dent de Toxodon. Pamp. moyen. (Arroyo del Medio). 5
73. Fragments de dents de Mastodon. 5

74. Une mâchoire supérieure et un fragment de mâchoire inférieure d'une espèce d'Auchenio. Pamp. sup. (Arroyo del Medio). 100
75. Un fragment de mâchoire de ruminant. Pamp. sup. (Pergamino). 20
76. Un Tibia de Milodon. Pamp. moyen. (Arroyo Pavon). 20
77. Une moitié de mâchoire inférieure de Glyptodon. Pamp. moyen. (Barranca del Parana, San Nicolas). 50
78. Un crâne de Milodon, incomplet. Pamp. moyen. (Arroyo del Medio). 500
79. Un crâne et un morceau de carapace de Glyptodon. Pamp. inf. (Barranca del Parana, San Lorenzò). 700
80. Un fragment de mâchoire inférieure de Megatherium. Pamp. inf. (Barranca del Parana, San Lorenzò). 30
81. Fragment d'un fémur. Pamp. moyen, San Lorenzò. 10
82. La partie antérieure d'un crâne de Scelidothérium. Pamp. moyen. (Barranca del Parana, San Lorenzò). 50
83. Une dent. Pamp. moyen. (Barranca del Parana, San Lorenzo). 5
84. Fragments de la mâchoire supérieure et inférieure d'une espèce de Gravigrades. Pamp. inf. (San Lorenzò). 30
85. Un morceau de fémur. Pamp. inf. (San Lorenzò). 10
86. Quelques fragments d'os calcinés. Pamp. moyen. (Barranca del Parana, San Lorenzò).
87. Un fragment de carapace d'une espèce de Biloricata. Pamp. inf. (Barranca del Parana, San Lorenzò). 5
88. Une dent de Mastodon. Pamp. moyen. (Barranca del Parana, San Lorenzò). 10
89. Un fragment de carapace de Hoplophorus. Pamp. sup. (Barranca del Parana, San Lorenzò). 20
90. Quelques fragments de dents de Mastodon. 5
91. Une dent d'une espèce de Loricata. Pamp. inférieur. (Barranca del Parana, San Lorenzò). 10
92. Quelqes plaques de carapace de Glyptodom. Pamp. inf. (Arroyo Pergamino). 10
93. Un fragment de crâne d'un Milodon, très endommagé. Pamp. moyen. (Arroyo Pavon). 150
94. Un fémur de Mastodon (quelques fragments qui appartiennent à ce fémur se trouvent dans la caisse No. 28). Pamp. sup. (Arroyo del Medio). 200
95. Un tibia et deux os du pied d'une grande espèce des Edentés. Pamp. moyen. (Arroyo del Medio.). 150

96. Un fragment de la carapace d'un Hoplophorus. Pamp. moyen. (Barranca del Parana, Rosario). 50

97. Un cubitus de Megatherium. Pamp. moyen. (Arroyo Pavon). 50

98. Un femur et un fragment de pelvis, provenant probablement d'un Macrauchenia. Pamp. moyen. (Arroyo Pavon). 300

99. Un fragment du crâne et une plalange d'un Megatherium. Pamp. moyen. (Arroyo Pergamino). 200

100. Un certain nombre de fragments de la carapace d'un Panochtus. Pamp. moyen. (Rio Carcaraña). 100

101. Quelques fragments de la carapace d'une espéce de Glyptodons. Pamp. inf. (Barranca del Parana, San Lorenzo). 50

102. Trois vertèbres cervicales provenant probablement d'un Palaeolama. Pamp. sup. (Victoria, Province d'Entre-Rios). 30

103. Une mâchoire inférieure d'un ruminant. Pamp. sup. (Baradero). 10

104. Deux os de pied provenant probablement d'un carnivore. Pamp. moyen. (Arroyo del Medio). 5

105. Quelques os des pieds de différents animaux. Pamp. moyen. (Barranca del Parana, San Nicolas). 10

106. Quelques os articulaires de ruminants. Pamp. moyen. (Barranca del Barana, Sanchez). 5

107. Un grand nombre de dents de ruminants. Pamp. moyen. (Barranca del Barana, Sanchez). 50

108. Quelques fragments de l'écaille d'une tortue. Pamp. moyen. (Arroyo Pergamino). 10

109. Une dent d'une grande espéce de Macrauchenia. Pamp. moyen. (Arroyo Ramallo). 10

110. Une incisive de Toxodon. Pamp. moyen. (Arr. Pergo.) 10

111. Un pied d'Equus pas tout à fait complet. Pamp. sup. (Arroyo Pergamino).. 50

112. Un radius d'un hoplopode (ongulé). Pamp. moyen. (Arroyo Pergamino). 20

113. Une dent d'une espèce d'Equus. Pamp. moyen. (Barranca del Parana. Sanchez). 5

114. Une phalange d'une espèce d'Equus. Pamp. moyen. (Barranca del Parana, Tonelero). 3

115. Un fragment de mâchoire de Toxodon. Pamp. moyen. (Barranca del Parana, Tonelero). 10

116. Un fragment de mâchoire supérieure de Scelidotherium. Pamp. sup. (Baradero). 20

117. Un fragment de carapace d'un Hoplophorus. Pamp. inf. (Barranca del Parana, San Lorenzò). 30

118. Quelques plaques de carapace de Glyptodon. Pamp. moyen. (Rio Salado). 10

119. Une phalange. (Entre Rios). 5

120. Un métacarpe de Palaeolama. Pamp. moyen. (Arroyo Pergamino). 10

121. Une écaille provenant probablement d'une tortue excessivement grande. Pamp. moyen. (Barranca del Parana, Tonelero). 50

122. Une grande partie du squelette avec le crâne d'un Scelidotherium. Pamp. sup. (San Nicolas). 3500

123. Un crâne avec mâchoire inférieure ainsi qu'une grande partie de squelette d'un Palaeolama. Pamp. inf. (Barranca del Parana, San Nicolas). 3500

124. Une grande partie du squelette et de la carapace d'un Hoplophorus. Pamp. sup. (Alverde près Rosario). 1500

125. Un crâne presque complet, un humérus, un radius, un cubitus, un tibia, 2 phalanges, un ongle, les deux côtes antérieures, 7 vertèbres dorsales, une vertèbre caudale, d'une espèce de Coelodon. Pamp. moyen. (Rio Carcaraña). 3000

126. Une moitié de la mâchoire inférieure et la partie antérieure de la mâchoire supérieure d'un jeune Scelidotherium. Pamp. inf. (Arroyo Pavon). 300

127. Une moitié de la mâchoire supérieure et une moitié de la mâchoire inférieure; quelques côtés, un tibia, et un os du pied d'un jeune Lestodon. Pamp. moyen. (Arroyo Pergamino). 400

128. Un fragment de la mâchoire inférieure d'un Milodon, formant la transition de cette espèce au Lestodon. 100

129. Un fragment de mâchoire d'une espèce de Palaeolama. Pamp. moyen. (Arroyo Pavon). 10

130. Un fragment de mâchoire de Scelidother. Pamp. sup. (Barranca del Parana, Baradero). 5

131. Un crâne et un mâchoire inférieure incomplets et quelques os de pied de Typotherium. Pamp. moyen. (Barranca del Parana, San Nicolas.) 800

132. Un humérus, un radius, un tibia, quelques os de pied et des plaques de carapace de Clamydotherium. Pamp. sup. (Pergamino). 250

133. Un fragment du crâne d'un Milodon. Pamp. moyen. (Arroyo Pergamino.) 100

134. Une mâchoire inférieure d'une espèce de Lama. Pamp. sup. (Baradero). 20

135. Une carapace incomplète, une queue et une partie du squelette d'un Glyptodon. Pamp. moyen. (Arroyo del Medio). 1000

136. Un mâchoire inférieure et une côte de Lestodon. Pamp. moyen. (Arroyo Pergamino.) 500

137. Un fragment de mâchoire d'une espèce de Lama. Pamp. sup. (Pergamino). 20

138. Une dent de Palaeolama. Pamp. infér. (Arroyo Pavon). 1

139. Une dent d'un grand ruminant. Pamp. inter. (Rio Carcarañа). 2

140. Un morceau de la mâchoire d'un carnivore. Pamp. moyen. (Arroyo del Medio). 20

141. Une mâchoire supérieure et une mâchoire inférieure d'un carnivore. Pamp. moyen. (Arroyo Pavon). 50

142. Un fragment de la mâchoire d'un carnivore. Pamp. sup. (Baradero). 20

143. Quelques dents d'un carnivore. Pamp. sup. (Barranca del Parana, Villa Constitucion). 10

144. Quelques dents de différents carnivores. Pamp. moyen. (Barranca del Parana, Sanchez). 10

145. Un fragment de mâchoire d'une espèce de Dicotyl. Pamp. moyen. (Arroyo del Medio). 10

146. Une dent d'Equus. Pamp. inférieure. (Barranca del Parana, San Lorenzò). 2

147. Un fragment de mâchoire d'Equus. Pamp. sup. (Baradero). 5

148. Une dent d'Equus. Pamp. moyen. (Arr. del Medio.) 2

149. Une dent d'Equus. Pamp. moyen. (Barranca del Parana, Sanchez). 2

150. Une dent d'Equus. Pamp. sup. (Arroyo Ramallo). 2

151. Un fragment de mâchoire d'Equus. Pamp. moyen. (Arroyo Pergamino). 5

152. Quelques fragments de carapace d'une espèce de Loricata. Pamp. moyen. (Arroyo del Medio). 5

153. Un os du pied et quelques plaques de la carapace d'une espèce de Loricata. Pamp. sup. (Pergamino). 10

154. Un certain nombre de plaques de carapace d'un Chlamydotherium. Pamp. moyen. (Barranca del Parana, Sanchez). 5

155. Un fragment d'une espèce de Loricata. Pamp. moyen. (Arroyo Ramallo). 20

156. Un fragment de carapace d'un Eutatus. Pamp. sup. (San Pedro). 2

157. Une dent d'un Milodon. Pamp. moyen. (Arroyo Pergamino). 10

158. Une plaque de carapace d'une espèce de Biloricata. Pamp. moyen. (Barranca del Parana Sanchez). 5

159. Un fragment de carapace d'un Hoplophorus. Pamp. moyen. (Rio Tercero). 10

160. Deux plaques de carapace d'une espèce de Loricata. Pamp. inférieur. (Arroyo dulce). 5

161. Quelques plaques de carapace d'un Chlamydotherium. Pamp. moyen. (Barranca del Parana, San Pedro). 5

162. Une dent de Milodon. Pamp. sup. (Baradero). 2

163. Une dent d'une espèce de Gravigrades. Pamp. moyen. (Arroyo del Medio). 2

164. Une dent de Milodon. Pamp. inf. (Arr. Pergamino). 2

165. Une dent de Scelidotherium. Pamp. inf. (Barranca del Parana, San Lorenzò.) 2

166. Une dent de Scelidotherium. Pamp. inf. (Arr. Pavon). 2

167. Une dent de Milodon. Pamp. moyen. (Arr. del Medio). 2

168. Une plaque de la carapace d'une espèce de Loricata. Pamp. sup. (Barranca del Parana, San Nicolas). 10

169. Une plaque de la carapace d'une espèce de Loricata. Pamp. sup. (Pergamino). 1

170. Une dent d'une petite espèce de Scelidotherium. Pamp. sup. (Pergamino). 5

171. Une dent de Scelidotherium. Pamp. sup. (Baradero). 2

172. Une dent probablement d'une espèce de Lestodon. Pamp. moyen. (Barranca del Parana, San Pedro). 2

173. Une dent d'un animal probablement du genre Milodon. Pamp. moyen. (Arroyo del Medio). 2

174. Une dent d'un Hoplophorus. Pamp. sup. (Pergamino). 2

175. Quelques dents d'Edentés de différentes couches de la formation pampéen. 5

176. Un fragment de crâne d'une très petite espèce de gravigrades. Pamp. inf. (Arroyo Pergamino). 20

177. Un fragment de mâchoire de Scelidotherium. Pamp. sup. (Baradero). 10

178. Une dent de Scelidotherium. Pamp. inf. (Barranca del Parana, San Lorenzò). 2

179. Une dent de Scelidotherium. Pamp. moyen. (Arroyo del Medio. 2

180. Un fragment de mâchoire inférieure probablement d'une petite espèce de Grypotherium. Pamp. sup. (Pergamino). 100

181. Une dent de Milodon. Pamp. moyen. (Arr. del Medio.) 2

182. Un fragment de mâchoire et quelques plaques de carapace d'une espèce très singulière de Loricata. Pamp. sup. (Arroyo Pergamino). 50

183. Une plaque de carapace d'un grand Hoplophorus. Pamp. inf. (San Nicolas). 2

184. Deux plaques de carapace d'une espèce de Loricata. Pamp. inf. (San Lorenzò). 5

185. Un fragment de mâchoire de Glyptodon. Pamp. sup. (San Nicolas). 10

186. Un os du pied d'un Hoplophorus. Pamp. moyen. (Arroyo del Medio.) 2

187. Un grand nombre d'os de pied d'Eutatus. Pamp. sup. (Pergamino). 50

188. Un fragment de mâchoire de Scelidotherium. Pamp. moyen. (Arroyo del Medio). 50

189. Un fragment de mâchoire de Milodon. Pamp. inf. (Arroyo Pergamino). 50

190. Une moitié d'une mâchoire inférieure de Grypotherium. Pamp. moyen. (Arroyo del Medio). 100

191. Une dent d'Equus. Pamp. moyen. (Barranca del Parana, Villa Constitucion). 2

192. Une dent d'Equus. Pamp. sup. (San Pedro). 2

193. Une dent d'Equus. Pamp. moyen. (Barranca del Parana, Sanchez). 2

194. Une dent d'Equus. Pamp. moyen. (Barranca del Parana, Sanchez). 2

195. Une dent d'Equus. Pamp. inf. (Ramallo). 2

196. Une dent d'Equus. Pamp. moyen. (San Nicolas). 2

197. Une dent d,Equus. Pamp. sup. (Pergamino). 2

198. Un radius et un métacarpe. Pamp. moy. (Pergamino.) 20

199. Une vertèbre dorsale. Pamp. inf. (Ramallo). 2

200. Une vertèbre cervicale. Pamp. moyen. (Arr. Pergo.) 2

201. Une vertèbre lombaire. Pamp. sup. (San Pedro). 2

202. Deux vertèbres lombaires. Pamp. inf. (Ar. del Medio). 2

203. Un fragment de mâchoire d'un ruminant. Pamp. sup. (Pergamino). 10

204. Une dent de ruminant. Pamp. moyen. (Barranca del Parana, Sanchez). 2

205. Une dent de Toxodon. Pamp. moyen. (Arr. Pergo.) 2

206. Une dent de Toxodon. Pamp. moyen. (Barranca del Parana, Sanchez). 2

207. Une dent d'une très petite espèce de Milodon. Pamp. moyen. (Arroyo Pergamino). 5

208. Une dent probablement d'une petite espèce de Toxodon. Pamp. moyen. (Barranca del Parana, Sanchez). 5

209. Une dent de Toxodon. Pamp. moyen. (San Nicolas). 5

210. Une dent de Toxodon. Pamp. inf. (Arr. Ramallo). 5

211. Une dent de Toxoden. Pamp. moyen. (Arr. Pavon). 5

212. Une dent de Toxodon. Pamp. moyen. (San Lorenzò). 5

213. Restes humains. Pamp. moyen. (Baradero). 3000

214. Une patte antérieure avec radius et cubitus, une vertèbre cervicale et deux vertèbres thorauques de Megatherium. Pamp. moyen. (Arroyo del Medio). 600

215. Un fragment de crâne; je croyais d'abord qu'il provenait d'un Panochtus mais je vois maintenant qu'il provient d'une espèce de Biloricata, dont le crâne n'est pas encore connu. Pamp. moyen. (Arr. Pergo.) 250

216. Un crâne avec machoire inférieure, quelques vertèbres cervicales d'une espèce de Biloricata. Pamp. infér. (San Nicolas). 3500

217. Un étui caudal de Doedicurus. Pamp. moyen. (Arroyo del Medio). 500

218. Un étui caudal de Panochtus. Pamp. moyen. (Arr. del Medio). 200

219. Un péroné d'un animal pas encore connu, appartenant aux Edentés. Pamp. inf. (Arroyo Pergamino). 100

220. Un fragment de crâne d'un Milodon. Pamp. moyen. (Arroyo del Medio). 100

221. Un fragment de mâchoire, probablement d'un Macrauchenia. Pamp. inf. (San Nicolas). 10

222. Un radius d'un ongulé. Pamp. moyen. (Arr. del Medio). 20

223. Un crâne, incomplet, une omoplate, un humérus, un radius avec cubitus, une patte antérieure entière, toutes les vertèbres cervicales, quelques vertèbres dorsales d'une très grande espèce de Macrauchenia. Pamp. moyen. (Arr. del Medio). 1510

224. Un crâne avec mâchoire inférieure, très bien conservé, un humérus, un radius, un cubitus, une patte antérieure, quelques vertèbres dorsales et thoraciques d'un Milodon. Pamp. inf. (Ar. del Medio). 3500

225. Une mâchoire inf. de Toxodon incomplète. Pamp. inf. (Barranca del Parana, Sanchez). 300

226. Un crâne et un fragment de carapace d'une espèce de Biloricata. Pamp. inf. (Bar. del Par., San Lorenzò). 500

227. Un crâne avec mâchoire inf. (je l'avais complètement réparé, mais il a été brisé en voyage) une partie d'un squelette et de la carapace d'un Eutatus. Pamp. sup. (Pergamino). 300

228. Un crâne incomplet et quelques plaques de la carapace d'un Eutatus. Pamp. infér. (Arr. Ramallo). 150

229. Une mâchoire infér. d'un Scelidotherium. Pamp. sup. (Sanchez). 100

230. Un radius d'une espèce d'Edentés. Pamp. infér. Arroyo Pergamino). 50

231. Une dent, une phalange, deux vertèbres caudales de Megatherium. Pamp. moyen. (Arr. Pergamino). 30

232. Un fragment de carapace de Glyptodon. Pamp. moyen. (Rio Salado). 10

233. Un fragment de mâchoire, une vertèbre cervicale d'un Hoplophorus. Pamp. sup. (Pergamino). 10

234. Quelques os de pied, probablement d'un rongeur. Pamp. sup. (Arroyo Pergamino). 5

235. Deux fragments de mâchoire d'un ruminant. Pamp. moyen. (Arroyo del Medio). 10

236. Une dent, probablement d'un Palaeolama. Pamp. sup. (Pergamino). 1

237. Une dent d'un ruminant. Pamp. inf. (Pergamino). 1

238. Une dent d'un ruminant. Pamp. moyen. (Arr. Pavon). 1

239. Une moitié d'une mâchoire inf. de ruminant. Pamp. inf. (Arroyo Ramallo). 20

240. Un dent d'un ruminant. Pamp. sup. (Baradero). 1

241. Fragment de mâchoire d'un ruminant. Pamp. sup. (Pergamino). 5

242. Deux fragments de mâchoire d'un ruminant. Pamp. moyen. (Arryo del Medio). 5

243. Un dent de ruminant. Pamp. inf. (Arr. Pergamino). 1

244. Un dent de ruminant. Pamp. sup. (Baradero). 1

245. Un dent de ruminant. Pamp. sup. (Pergamino). 1

246. Fragment de mâchoire supér. et de mâchoire inf. d'un carnivore. Pamp. moyen. Villa Constitucion. 40

247. Quelques fragments de la carapace d'une tortue. Pamp. moyen. (Pergamino). 10

248. Une dent, probablement d'un Macrauchenia. Pamp. moyen. (Villa Constitucion). 10

249. Une dent de Toxodon. Pamp. moyen. (Arr. del Medio). 5

250. Deux dents de Toxodon. Pamp. moyen. (Ar. Pavon). 5

251. Une dent d'Equus. Pamp. moy. (Bar. d. Par., Sanch.) 2

252. Un crâne incomplet, un fémur et quelques vertèbres dorsales d'un rongeur. Pamp. moyen. (San Nicolas.) 50

253. Un crâne avec mâchoire inférieure incomplet de Laogostomus. Pamp. sup. (Pergamino). 20

254. Deux mâchoires infér. de Laogostomus. Pamp. moyen. (Villa Constitucion). 20

255. Fragment de mâchoire, infér. de Laogostomus. Pamp. moyen. (Villa Constitucion). 5×

256. Fragment de mâchoire infér. de Miopotamus. Pamp. inf. (San Pedro). 5

257. Fragment de mâchoire infér. d'une espèce de Cavia. Pamp. sup. (Pergamino). 5

258. Humérus d'un ongulé. Pamp. sup. (Pergamino). 10

259. Fémur d'une très grande espèce de Loricata. Pamp. inf. (Arr. Pergamino). 100

260. Fragment de carapace d'une espèce de Biloricata (ce fragment appartient probablement à une espèce du même genre que le crâne No. 216. J'avais déjà trouvé auparavant un fragment de crâne avec un fragment de carapace présentant les mêmes particularités. Pamp. moyen. (Villa Constitucion). 20

261. Une phalange probablement d'un Hoplophorus. Pamp. sup. (Pergamino). 5

262. Fragment de mâchoire de Laogostomus. Pamp. moyen. (Arr. Pavon). 5

263. Crâne de Ctenomys. Pamp. sup. (Rio Segundo). 10

264. Fragment de mâchoire infér. d'un rongeur. Pamp. sup. (Rio Segundo). 5

265. Deux fémurs, un tibia, un vertèbre dorsale d'un rongeur. Pamp. sup. (Pergamino). 10
266. Quelques os de différ. animaux. Pamp. sup. (Sanchez). 10
267. Une phalange d'une petite espèce de Biloricata. Pamp. moyen. (Arroyo dulce). 2
268. Quelques dents de rongeurs. Pamp. moy. (Sanchez). 5
269. Quelques dents provenant de différ. couches du Pamp. 5
270. Deux dents des espèce de pachydermes. Pamp. moyen. (Sanchez). 10
271. Quelques dents. Pamp. sup. (Pergamino). 5
272. Quelques os isolés provenant de différentes couches de la form. pamp. 5
273. Quelques dents de poissons. Pamp. moyen. (Sanchez). 5
274. Quelques fragments d'os, provenant de l'ancien lit d'un fleuve. Pamp. moyen. (Sanchez). 5
275. Quelques vertèbres dorsales d'Eutatus. Pamp. sup. (San Nicolas). 50
276. Quelques dents de Scelidotherium. Pamp. sup. (San Nicolas). 50
277. Une vertèbre dorsale. Pamp. moyen. (Rio Carcaraña) et une arcade zygomatique de Scelidotherium. Pamp. moyen. (Arroyo del Medio). 20
278. Un crâne avec la mâchoire inf. aussi qu'une partie du squelette et de la carapace d'une espèce d'Hoplophorus (pas bien conservé). Pamp. moyen. (Arroyo del Medio). 100
279. Grande partie d'un squelette de Megatherium, il manque, entre autres, le crâne. Pamp. moyen. (Arr. del Medio). 6000
280. Un crâne avec la mâchoire inf. ainsi qu'une grande partie du squelette d'un Scelidotherium. Pamp. moyen. (Arr. del Medio.) 3000
281. Crâne avec la mâchoire inférieure ainsi qu'une grande partie d'un squelette de Scelidotherium. Pamp. moyen. (Pergamino). 3500
282. Mâchoire inférieure d'un Mastodon. Pamp. moyen. (Arroyo del Medio). 500
283. Un squelette tant complet de Glyptodon et la carapace du même animal. Pamp. moyen. (Ar. Sepedo.) 8000
284. Fragment de carapace d'un Glyptodon. Pamp. moyen. (Arroyo Pergamino). 200
